# Cuticular Hydrocarbons‐Revealed Functional Groups and Seasonal Acclimation in Sympatric Fig Wasp Mating Assemblages

**DOI:** 10.1002/ece3.73976

**Published:** 2026-07-07

**Authors:** Hua Xie, Shouxian Zhang, Yonghui Zhu, Subo Shao, Pei Yang, Zongbo Li, Yuan Zhang

**Affiliations:** ^1^ Key Laboratory of Forest Disaster Warning and Control in Yunnan Province Southwest Forestry University Kunming Yunnan China; ^2^ College of Traditional Chinese Medicine Yunnan University of Chinese Medicine Kunming Yunnan China; ^3^ Key Laboratory for Forest Resources Conservation and Utilization in the Southwest Mountains of China Southwest Forestry University Kunming Yunnan China

**Keywords:** cuticular hydrocarbons, fig–wasp symbiosis, functional groups, mate attractiveness, seasonal plasticity

## Abstract

Interspecific mating can increase when sympatric species mature simultaneously, posing challenges for mate recognition and resource utilization. Cuticular hydrocarbons (CHCs) contribute to desiccation resistance and act as chemical signals in communication. However, it is not sufficiently understood how the CHC traits of sympatric species respond to overlapping ecological, reproductive, and seasonal pressures. This study examines variation in cuticular hydrocarbons among five *Ficus semicordata*‐associated fig wasp species and explores potential links between CHC profiles, mate recognition, and seasonal environmental variation, complemented by male mate‐choice assays in two species. Species‐specific CHC profiles with sexual dimorphism, combined with male mate choice, are consistent with a potential chemical basis for mate recognition. Species that share resource acquisition strategies (functional groups) tend to exhibit similar CHC patterns. The CHC composition adjusted seasonally among the four dominant species, and these patterns varied among functional groups and between sexes. This indicates that even under shared host and comparable macroclimatic conditions, species and sexes maintain distinct chemical profiles through adjustments in the composition and proportions of CHC. These findings provide a foundational framework for predicting how chemical traits mediate ecological adaptation and speciation in coexisting insect communities under spatiotemporal pressures.

## Graphical abstract

This study demonstrates how climate‐driven seasonal variation shapes the chemical phenotypes of five sympatric fig wasp species. We show that cuticular hydrocarbons (CHCs) are sex‐ and species‐specific signals linked to male mate choice and functional guilds, revealing a chemical basis for niche differentiation within a shared host system. Importantly, CHCs exhibit adaptive, non‐uniform plasticity under warmer and drier conditions, highlighting how environmental filtering can restructure chemical phenotypes and interspecific interaction networks.

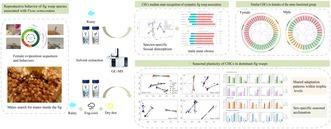

## 1 Introduction

In multi‐species social or ecological environments, individuals often face perceptual challenges in identifying suitable mates, requiring the extraction of species‐discriminative cues (e.g., chemical, physical, or visual signals) from overlapping stimuli amid concurrent sensory noise (McDermott 2009; Wyatt 2014; Blomquist and Ginzel 2021). This “signal interference” problem is particularly pronounced in chemical communication systems—when multiple species release partially overlapping compounds, receivers must detect species‐specific signals from complex chemical backgrounds. Such interference exerts strong selective pressures favoring the evolution of highly specific communication signals (Ercit and Gwynne 2015). This evolutionary pressure is particularly acute in sympatric insect communities forming dense reproductive swarms, where timely mate recognition directly determines sexually reproductive success (Krupke et al. 2011; Little et al. 2024). Such multispecies mating assemblages occur widely across taxa, including bark beetle aggregations (Gomez et al. 2023), mixed‐species congregations of mycophagous drosophilids (Toda et al. 1999), malaria mosquitoes (*Anopheles* spp.) (Diabaté et al. 2006), and fig wasps (Segar et al. 2025).

Among communication systems, cuticular hydrocarbon (CHC) profiles represent evolutionary stability and information‐rich signals (Wyatt 2014; Chung and Carroll 2015; Blomquist and Ginzel 2021). Cuticular hydrocarbons serve dual functions: preventing water loss through a hydrophobic barrier and allowing their function as contact pheromones, while conveying critical information about nest origin, age, caste, sex, and reproductive status (Kather and Martin 2015; Sprenger and Menzel 2020; Blomquist and Ginzel 2021; Yun et al. 2023; Bell et al. 2024), with structural features determining their biological roles (Gibbs and Pomonis 1995; Blomquist and Ginzel 2021). Despite extensive knowledge of CHCs' dual roles in environmental adaptation and chemical communication (Gibbs and Pomonis 1995; Wyatt 2014; Berson Jacob et al. 2019; Blomquist and Ginzel 2021), the specific mechanisms basis of how insects maintain the specificity and reliability of chemical signals under environmental fluctuations—particularly within densely aggregated sympatric species communities—remains poorly understood.

Comparative analyzes demonstrate stronger CHC divergence in sympatric versus allopatric sister taxa with equivalent genetic distances, highlighting intensified selection for signal specificity under coexistence (Noor 1999; Lukhtanov et al. 2005). This chemical specialization facilitates adaptive radiation while maintaining species integrity (Mullen and Shaw 2014). Furthermore, *Drosophila suzukii* exhibits seasonal CHC plasticity as an adaptive strategy (Kárpáti et al. 2023). These is evidence that CHCs are not exclusively shaped by sexual selection; rather, their composition is dynamically regulated in response to environmental variables (temperature, humidity, diet) and biological factors (geographic origin, genetic background, developmental stage, interspecific competition) (Sprenger and Menzel 2020; Wittke et al. 2022; Yun et al. 2023). Thus, CHCs must simultaneously fulfill two potentially conflicting demands: maintaining signal reliability for communication while adapting to environmental variability for survival. How this balance is achieved remains unclear in sympatric, seasonally fluctuating systems. In fig wasps, CHCs are known to mediate multitrophic interactions (e.g., chemical camouflage against ants) (Ranganathan et al. 2015; Wang et al. 2018), but studies have focused mainly on cross‐trophic or defensive functions, with less attention to chemical signal differentiation among sympatric species. Multiple fig wasp species coexist at high densities and develop synchronously inside the enclosed syconium, generating a chemically complex environment with signal overlap. Consequently, how chemical signals maintain specificity and stability under environmental variation and multispecies interference remains poorly understood.

The fig wasp community represents an ideal natural system to explore signal differentiation under sympatric coexistence (Segar et al. 2025). In the fig‐fig wasp mutualism system, fig wasps develop within galled flowers of enclosed syconia. A single syconium may host multiple wasp species, including typically one to four pollinator species and ranging from 1 to 30 non‐pollinating wasp species (Cook and Segar 2010). Species richness may vary substantially among *Ficus* lineages (e.g., section), fig size, and ecological contexts (Borges 2015, 2021). These species belong to three functional groups: (1) gall‐makers (pollinators and non‐pollinators), which are further divided into early gallers that oviposit in very young figs and induce galls in the receptacle tissue, and ovary gallers that oviposit during the receptive phase and induce galls within the ovules; (2) kleptoparasites, which exploit pre‐existing galls; and (3) parasitoids, which parasitize other wasp larvae (Bronstein 1999; Borges 2015; Barros et al. 2026). However, their occurrence varies across syconia: pollinating gall‐makers are almost always present, whereas non‐pollinating gallers, kleptoparasites, and parasitoids occur less frequently, and their presence depends on fig species and ecological context. Parasitoids colonize syconia sequentially after gall‐makers and kleptoparasites, relying on species‐specific chemical cues for host localization (Proffit et al. 2007). They share nearly identical ecological niches and developmental schedules while maintaining strict reproductive isolation (Krishnan et al. 2014; Yadav et al. 2018; Liu et al. 2019). Moreover, the marked sexual dimorphism in fig wasps reflects distinct life‐history roles: wingless, sensory‐reduced males remain confined to the fig cavity, inseminating females or releasing them post‐mating, while winged females disperse to oviposit (Cook et al. 1997; Xiao et al. 2013). Within this complex and dynamic environment, early‐maturing males face intense competition to locate conspecific females. Specialized signaling mechanisms, likely species‐specific CHCs (Partan et al. 2005) or substrate‐borne vibrations (Hebets and Papaj 2005), are critical for mate localization. Airborne sound detection is improbable given the absence of auditory structures in fig wasps (Elias et al. 2018; Liu et al. 2019). Instead, studies have shown that male fig wasps rely on female CHCs as effective contact sex pheromones for mate recognition, even when females remain hidden within galls (Krishnan et al. 2014; Yadav et al. 2018; Liu et al. 2019). This ecological scenario creates a chemically crowded environment in which individuals must rely on species‐specific cues to locate and recognize mates.

Building on (Xie et al. 2024), which documented sexual dimorphism and seasonal variation in *Ceratosolen gravelyi* CHCs, we analyzed CHC profiles of five sympatric *Ficus semicordata*‐associated fig wasp species across genera and functional groups under distinct seasonal conditions. Our objectives were to: (1) characterize interspecific and intraspecific sexual differentiation in CHC compositions, (2) test whether males show directional preference for conspecific female CHCs and whether this preference is species‐specific, and (3) evaluate whether seasonal plasticity in CHCs contributes to maintaining signal reliability under fluctuating environmental conditions. Through this integrative approach, we tested the hypothesis that CHC diversification provides a chemical solution to the “signal interference challenge” faced by sympatric fig wasps, yet realized through molecular and behavioral adaptations.

## 2 Materials and Methods

### 2.1 Study Sites and *Ficus* System

We collected predispersal male syconia of *Ficus semicordata* from the Xishuangbanna Tropical Botanical Garden (21.92°–22.06° N, 101.16°–101.29° E), Yunnan, China, and surrounding areas. This region exhibits a tropical monsoon climate with three seasons: dry‐hot (March–May), rainy (June–October), and fog‐cool (November–February). *F. semicordata* thrives in forest edges, roadsides, and valleys. Male syconia host five fig wasp species with extreme sexual dimorphism: winged females disperse for oviposition, while wingless males remain confined, specializing in conspecific mate location. The sole pollinator, *Ceratosolen gravelyi*, enters female‐phase syconia to pollinate and oviposit, while four non‐pollinators with different ecological strategies, including the gall‐associated species *Philotrypesis dunia*, the kleptoparasite *Sycophaga cunia*, and the parasitoids *Apocrypta* sp. and *Sycoscapter trifemmensis*, oviposit externally (Kerdelhué and Rasplus 1996; Compton et al. 2018). Non‐pollinators colonize sequentially post‐floral transition, with overlapping oviposition periods between *Apocrypta* sp. and *S. trifemmensis* (for details, see Figure S1).

All harvested syconia were enclosed in fine‐mesh bags and transported to the lab. Following natural wasp emergence, syconia were frozen at −20°C to induce temporary paralysis and immobilization of fig wasps, enabling prompt collection, taxonomic sorting, and preparation for subsequent experimental procedures.

### 2.2 Extraction and Analysis of Cuticular Hydrocarbons

CHCs from both sexes of *F. semicordata* sympatric fig wasps were extracted via solvent immersion. Males and females were separated prior to extraction based on morphology, with females being winged and males wingless; male individuals were further identified to species under a stereomicroscope. For each species and sex, 50 adult wasps were pooled into a 2 mL glass vial (Agilent Technologies, USA) as one biological replicate and washed twice with 200 μL n‐hexane. Combined extracts were concentrated to 48 μL under nitrogen flow and transferred to a 400 μL insert‐equipped vial (Agilent Technologies, USA). Subsequently, 1 μL of each internal standard (n‐tetradecane and n‐hexadecane, 2.5 mg/μL) was added, resulting in a final volume of 50 μL for absolute quantification. A total of 230 samples were processed, with seasonal replicates (rainy, fog‐cool, dry‐hot) limited to 10 male–female pairs per species. Sixty of these samples (CHC data for *C. gravelyi* across seasons) were provided by Xie et al. (for details, see Table S1). Partial datasets included fewer replicates due to seasonal availability, and *P. dunia* specimens were exclusively collected during the rainy season.

Each samples (1 μL) was analyzed using an Agilent 7890B GC system equipped with an HP‐5MS Inferno column (30 m × 0.25 mm, ID 0.25 μm film). Injector temperature was 250°C (split mode 10:1), with helium carrier gas at 1.0 mL/min. Oven protocol: 40°C (2 min), 3°C/min to 150°C, 8°C/min to 260°C (5 min hold), 20°C/min to 300°C (10 min hold). Effluents were analyzed via Agilent 5977A MSD (70 eV ionization; ion source 230°C; transfer line 250°C). Compounds were identified using NIST 2008/2017 spectral libraries, retention time matching, Kováts retention indices, and NIST WebBook references (https://webbook.nist.gov) (Kováts 1965; Carlson et al. 1989). Retention indices (RI) were calculated using a C₆–C₄₀ n‐alkane series. Quantitative analysis followed internal standardization using the formula: *C*_x_ = *A*_x_/*A*_IS_ × *C*_IS_, where *C*_x_ represents the concentration of the target compound, *A*_x_ and *A*_IS_ are the peak areas of the target compound and the internal standard, respectively, and *C*_IS_ is the known concentration of the internal standard (Aitchison 1986).

### 2.3 Behavioral Bioassays

Behavioral assays quantified male short‐range orientation toward female CHCs, reflecting the initial step of mate recognition rather than mating itself. Among the five sympatric species examined, only males of *C. gravelyi* and *S. trifemmensis* exhibited sufficient abundance and locomotor activity to allow for reproducible and statistically robust behavioral testing. The remaining three species were less abundant within the syconia or displayed weak activity after emergence, making it difficult to obtain sufficient numbers of active males for quantitative assays. Therefore, the two selected species represented experimental feasibility rather than behavioral bias. Individual males were placed on a 10 cm agarose‐coated glass plate (1% gel) flanked by two stimulus females (3 mm apart) (Krishnan et al. 2014), reflecting short‐range orientation toward female CHCs. Males were assigned to two groups: (1) conspecific female vs. heterospecific female, and *Apocrypta* (2) untreated (intact CHCs) vs. hexane‐washed females (CHC‐removed). All females were immobilized by brief cryo‐treatment (−80°C for 5 min) prior to assays to prevent movement while maintaining intact CHC profiles, except for those subjected to CHC removal. For CHC removal, females were washed in 500 μL n‐hexane with vortexing for 10 min. Trials allowed 5 min responses, with stimulus positions randomized (46–81 replicates/condition) to eliminate spatial bias (see Table S2 for full details). Males were used once to prevent habituation.

### 2.4 Morphometric Analysis of Fig Wasp Taxa

Following n‐hexane extraction, fig wasps were photographed using a Carl Zeiss stereomicroscope (Model V20, Germany). Morphometric measurements, including body length (measured from the vertex of the head to the abdominal segment containing the external genitalia), head length, head width (interocular distance), hind tibia length, and ovipositor length were conducted using Image J software (Version 1.62, https://imagej.nih.gov). The study analyzed 720 specimens in total, comprising 30 male and 30 female individuals per species per season.

### 2.5 Data Analyses

CHCs were sampled across three seasons, but *P. dunia* absent from collections in the dry‐hot and fog‐cool seasons, a limitation potentially linked to seasonal shifts in fig wasp community structure (Aung et al. 2022), analyses unrelated to seasonal variation utilized CHC profiles solely from the rainy season dataset to ensure comparability among species. Furthermore, compounds constituting < 0.1% of total abundance were excluded from the analyses to mitigate confounding effects of seasonal plasticity on CHC composition.

To visualize interspecific variation in CHC profiles across five fig wasp species, we conducted non‐metric multidimensional scaling (NMDS) using Bray–Curtis dissimilarity matrices (Oksanen et al. 2016). Interspecific vs. intraspecific differences were tested using PERMANOVA, and key hydrocarbons contributing to species differentiation were identified using random forest models. Hierarchical clustering assessed chemical signature alignment with functional groups in male/female profiles. For seasonal CHC comparisons in four dominant species ( *C. gravelyi* , *S. cunia*, *Apocrypta* sp., *S. trifemmensis*), we analyzed compounds meeting: (1) relative abundance > 5% or (2) high random forest feature importance. These were categorized into structural classes (n‐alkanes, methylalkanes, alkenes, methylalkenes) and evaluated using Kruskal–Wallis tests with Nemenyi post hoc tests (Dinno 2024). The effects of climate variables (mean seasonal temperature and cumulative seasonal precipitation) on CHC composition were assessed using PERMANOVA based on Euclidean distance matrices, with separate models for each hydrocarbon class and total CHC content (Benjamini and Hochberg 2018). Principal component analysis (PCA) was used as an exploratory approach to visualize whether seasonal clustering in morphometric traits paralleled the CHC‐based seasonal patterns. Male behavioral responses were analyzed using Fisher's exact test, followed by McNemar's test with FDR correction.

All analyses were performed in R v4.0.2 (https://cran.r‐project.org/); figures were generated in GraphPad Prism v8.0.2 (Boston USA, https://www.graphpad.com). Detailed analytical procedures and parameter settings are provided in the Supplementary Methods section of the Supporting Informations. All data involved in the analysis are provided in a publicly available dataset (see Data Availability Statement).

## 3 Results

### 3.1 Species‐Specific and Sexual Dimorphic Patterns of CHC Profiles in Sympatric Fig Wasps

**FIGURE 1:**
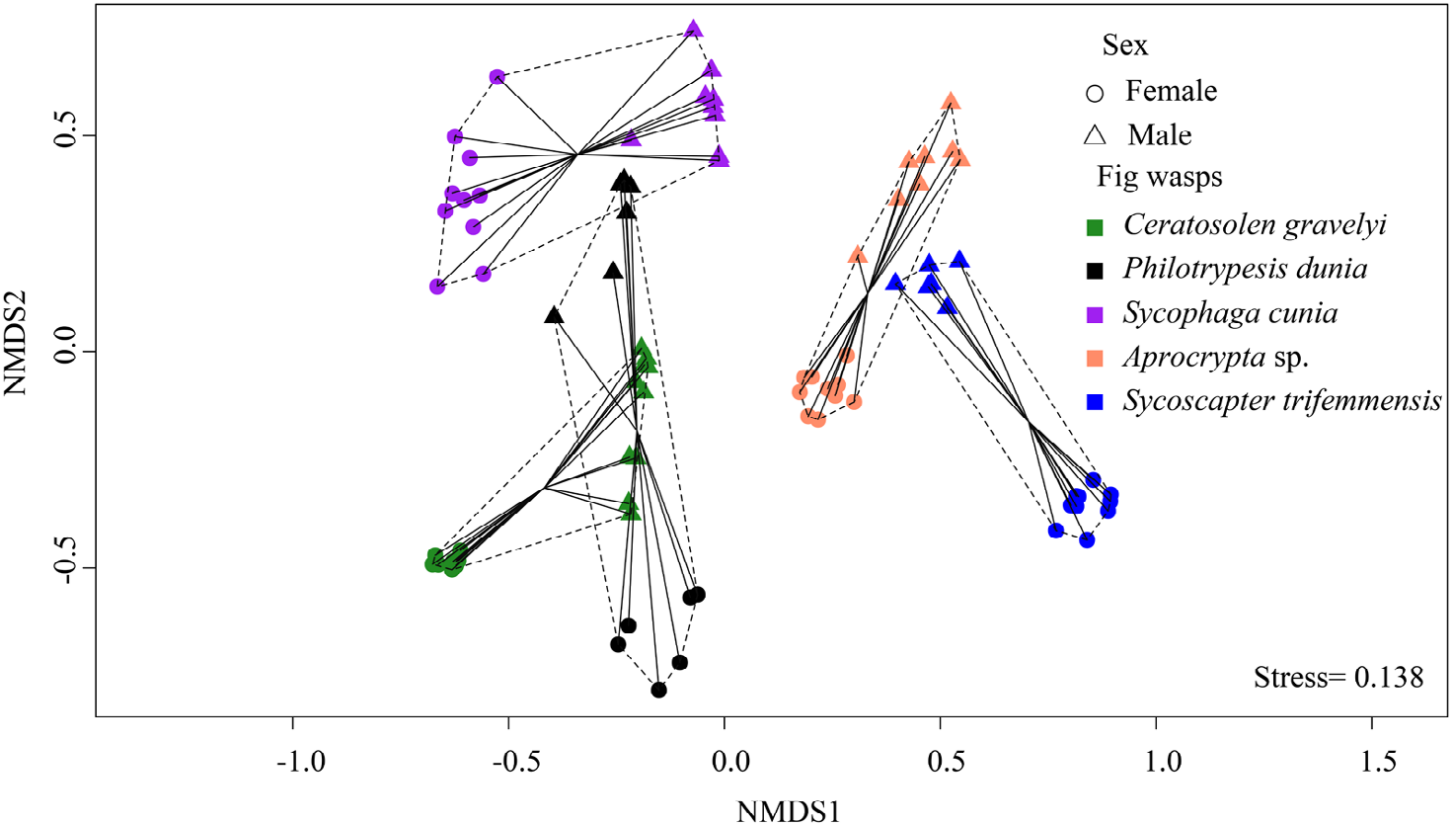
Non‐metric multidimensional scaling (NMDS) ordination of cuticular hydrocarbon profiles in fig wasps associated with *Ficus semicordata*.

We identified 42 CHC compounds across five *F. semicordata*‐associated fig wasp species, comprising n‐alkanes, branched alkanes, alkenes, and branched alkenes (C13–C44). n‐alkanes dominated (> 50% total CHCs) in all species, with significant interspecific and intersexual variation in composition and abundance (Table S3). NMDS ordination analysis revealed clear species segregation (Figure 1), which was statistically confirmed by PERMANOVA (global *R* = 0.973, *p* < 0.001; pairwise *R* > 0.176, *p* < 0.004; Table S4), despite partial overlap between species, supporting species‐specific signatures and sexual dimorphism. Random forest models identified six key compounds (15‐Me‐C29, n‐C35, n‐C27, 17‐C35:1, 2‐Me‐C28, n‐C34) critical for differentiation (Table S3). The number of variables (*n* = 6) was determined using 10‐fold cross‐validation (five repeats) with minimal error (Figure S2).

### 3.2 Hierarchical Cluster Analysis of CHC Profiles in *F. Semicordata*‐Associated Fig Wasps

**FIGURE 2:**
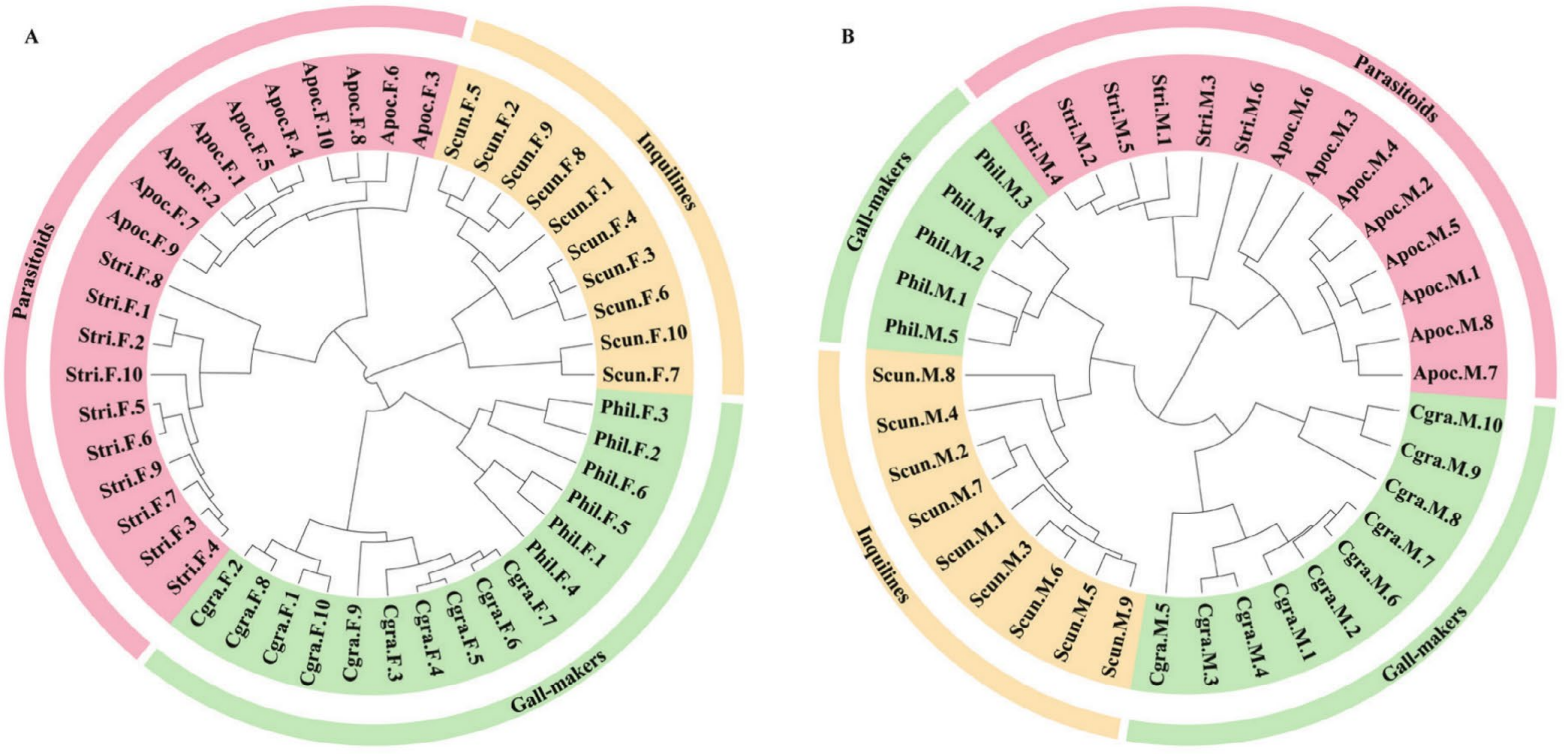
Hierarchical clustering analysis of cuticular hydrocarbon profiles in females (A) and males (B) of five sympatric fig wasp species: *Ceratosolen gravelyi* (Cgra; females *n* = 10, males *n* = 10), *Philotrypesis dunia* (Phil; females *n* = 6, males *n* = 5), *Sycophaga cunia* (Scun; females *n* = 10, males *n* = 9), *Apocrypta* sp. (Apoc; females *n* = 10, males *n* = 8), and *Sycoscapter trifemmensis* (Stri; females *n* = 10, males *n* = 6).

NMDS ordination revealed distinct spatial clustering patterns in CHC profiles among the five fig wasp species associated with *F. semicordata*. Specifically, *P. dunia* exhibited close CHC profile proximity to *C. gravelyi* , followed by *S. cunia*, while *Apocrypta* sp. clustered near *S. trifemmensis*. *Apocrypta* sp. and *S. trifemmensis* showed maximal CHC divergence from the other three species (Figure 1). This spatial configuration suggests potential alignment between CHC signatures and fig wasp functional group classifications. Hierarchical clustering analysis corroborated this hypothesis, demonstrating robust aggregation of CHC profiles among conspecific females within functional groups, a pattern absent in males (Figure 2). However, these clustering patterns exhibited seasonal instability, with no clear functional group associations observed across dry‐hot and fog‐cool seasons (Figures S3–S6).

### 3.3 Seasonal Dynamics of CHC Profiles in Dominant Fig Wasps Associated With *F. Semicordata*

**FIGURE 3:**
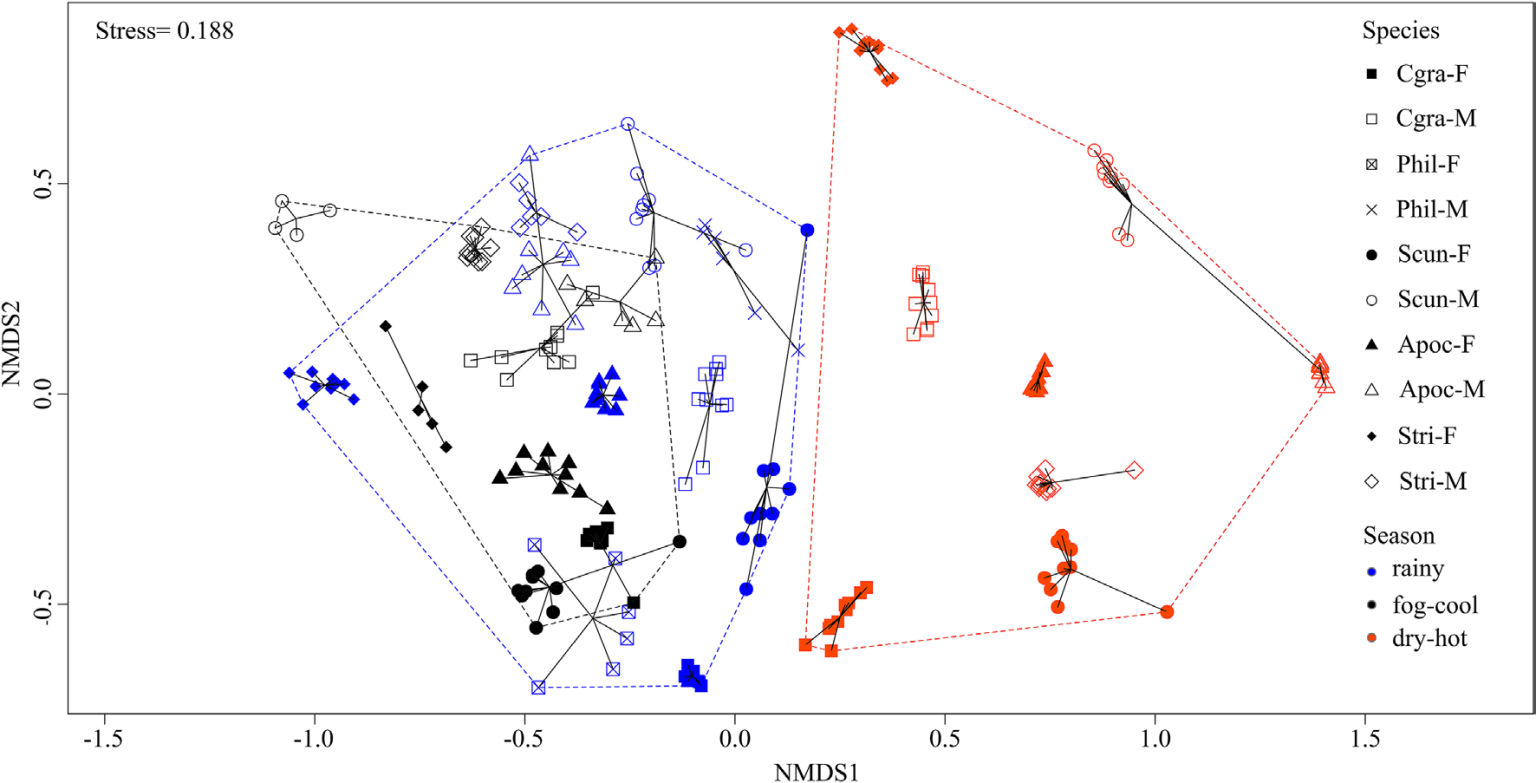
Non‐metric multidimensional scaling (NMDS) ordination of cuticular hydrocarbon profiles in dominant fig wasp species associated with *Ficus semicordata* across seasonal variations. Points represent individual samples, coloured by season (rainy, fog‐cool, dry‐hot) and shaped by species–sex categories.

A comprehensive analysis of CHC profiles across three seasons revealed substantial interspecific and seasonal differences in both qualitative composition and quantitative abundance among four dominant *F. semicordata*‐associated fig wasp species (Table S5). These findings highlight pronounced seasonal plasticity in CHC expression. NMDS ordination demonstrated clear seasonal clustering: fog‐cool and rainy season profiles showing spatial proximity, while dry‐hot season profiles formed distinct clusters separate from other seasons (Figure 3; Figure S7). Sexual dimorphism in CHC signatures remained stable across all seasonal conditions for all four species (Figure S7; Table S5). Hierarchical clustering (Figure S8) further confirmed this pattern, with fog‐cool and rainy seasons clustering together in both sexes, and dry‐hot season forming a separate branch (less pronounced in males).

**FIGURE 4:**
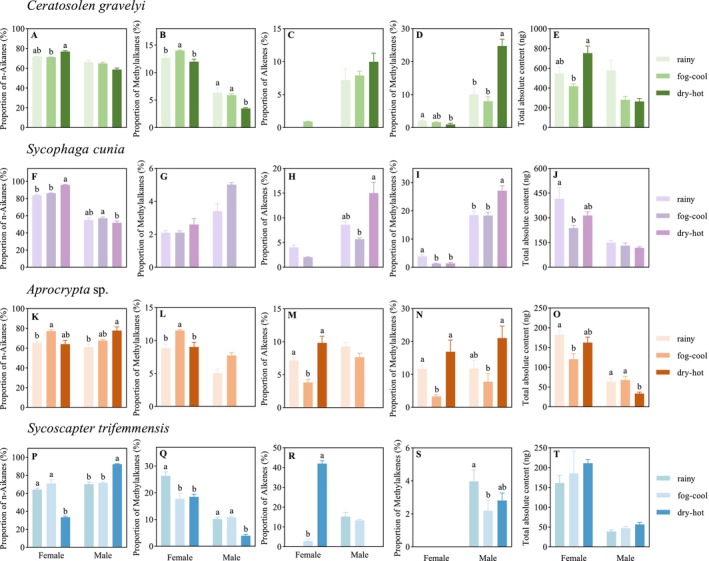
Seasonal effects on cuticular hydrocarbon profiles of male and female dominant fig wasps associated with *Ficus semicordata* (mean ± SE). Different lowercase letters denote significant differences between seasons (*p* < 0.05).

Species‐specific seasonal patterns emerged in CHC composition. *C. gravelyi* females exhibited elevated n‐alkane (n‐C34, n‐C36, n‐C40, n‐C44) proportions and total CHC content during dry‐hot seasons compared to fog‐cool periods (Figure 4A,E), while methylalkane 2‐Me‐C28 decreased in dry‐hot and rainy seasons. Males showed reduced methylalkanes but increased methylalkenes (squalene) during dry‐hot conditions (Figure 4B,D). PERMANOVA identified temperature and precipitation as primary drivers of n‐alkane variation and total CHC content in both sexes, with precipitation governing methylalkane dynamics and temperature regulating male‐specific methylalkene changes (Table S6).

*Sycophaga cunia* displayed sex‐divergent responses: females showed dry‐hot season peaks in n‐alkanes (n‐C34, n‐C36, n‐C40, n‐C44) (Figure 4F) and rainy season maxima for squalene (Figure 4I), whereas males exhibited fog‐cool season n‐alkane dominance versus dry‐hot peaks in alkenes (17‐C35:1) and methylalkenes (squalene) (Figure 4F,H,I). Male 2‐Me‐C28 became undetectable in dry‐hot seasons (Figure 4G). Environmental regulation differed by sex; female CHCs responded to combined temperature/precipitation effects for most compounds, while male profiles showed precipitation‐driven n‐alkane/alkene variations and dual‐factor methylalkane regulation (Table S6).

*Apocrypta* sp. females demonstrated fog‐cool season maxima for n‐alkanes (n‐C34, n‐C35, n‐C36, n‐C40, n‐C43, n‐C44) and 2‐Me‐C28 (Figure 4K,L), contrasting with dry‐hot peaks in 17‐C35:1 and squalene (Figure 4M,N). Total CHC content peaked during rainy seasons (Figure 4O). Males exhibited inverse patterns: dry‐hot season elevations in n‐alkanes (n‐C34, n‐C36, n‐C44) and methylalkenes (squalene) (Figure 4K,N), with undetectable methylalkanes/alkenes during this period (Figure 4L,M). Environmental drivers included dual temperature/precipitation effects on female n‐alkanes/alkenes/methylalkenes versus precipitation‐dominated regulation of male n‐alkanes/alkenes (Table S6).

*Sycoscapter trifemmensis* females showed reduced dry‐hot season n‐alkanes (n‐C29, n‐C31, n‐C34, n‐C35, n‐C36, n‐C44) (Figure 4P) and minimal 2‐Me‐C28 across seasons (Figure 4Q), with alkenes (C29:1, 17‐C35:1) exceeding 40% abundance in dry‐hot conditions but absent in rainy periods (Figure 4R). Males displayed dry‐hot season n‐alkane peaks (Figure 4P) and suppressed methylalkanes (Figure 4Q). Regulatory patterns involved dual temperature/precipitation effects on female n‐alkanes versus precipitation‐dominated control of male total CHC content and female alkenes (Table S6).

### 3.4 Behavioral Bioassays

**FIGURE 5:**
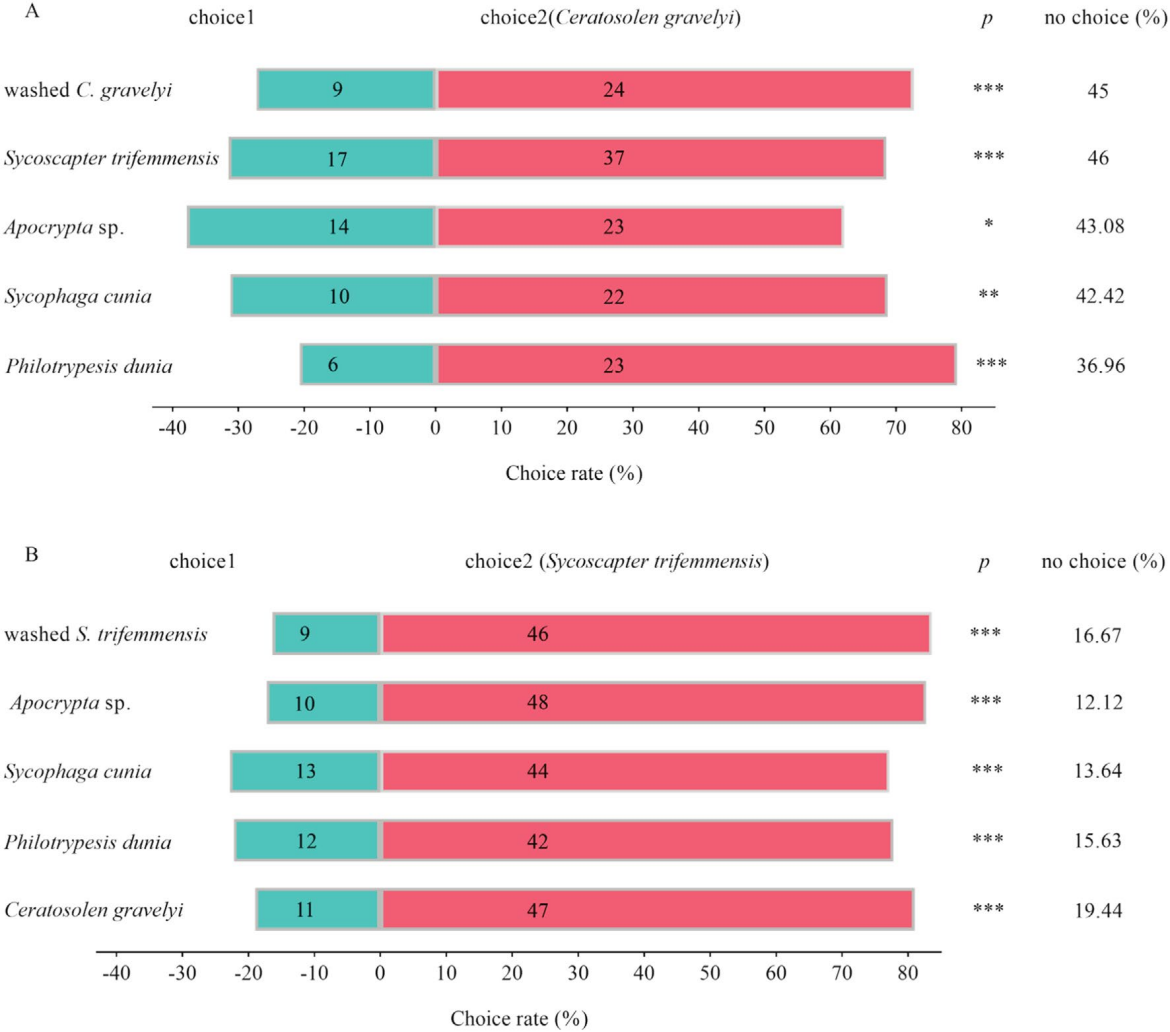
Two‐choice behavioral assay with males of *Ceratosolen gravelyi* (A) and *Sycoscapter trifemmensis* (B) exposed to conspecific vs. heterospecific female chemical cues. Non‐responding males within the 5 min observation period were excluded from subsequent chi‐square analysis. Bar heights indicate the number of males selecting each stimulus. *p < 0.05, **p < 0.01, ***p < 0.001; N denotes total number of tested males.

Male *C. gravelyi* and *S. trifemmensis* exhibited species‐specific orientation toward conspecific females over heterospecific or CHC‐removed conspecifics (Figure 5). *C. gravelyi* males exhibited notably higher non‐selection rates than *S. trifemmensis*, indicating interspecific differences in responsiveness during the behavioral tests.

## 4 Discussion

### 4.1 Cuticular Hydrocarbons as Potential Cues for Mate Recognition Among Sympatric Fig Wasp Species

Accurate mate recognition via species‐specific CHCs is crucial for preventing hybridization in densely interacting insect communities. Our study of five sympatric *F. semicordata*‐associated fig wasp species revealed sexually dimorphic and highly species‐specific CHC profiles, consistent with their potential role in reproductive isolation and reflecting strong genetic determination (Buellesbach et al. 2013; Zhang et al. 2014; Kather and Martin 2015; Shahandeh et al. 2018; Boff and Ayasse 2024; Narah et al. 2024). Behavioral assays showed that males of *C. gravelyi* and *S. trifemmensis* significant preferences for conspecific females with intact CHC profiles, indicating that CHCs may be function as short‐range recognition cues. As behavioral assays were limited to two species and used freeze killed females, which may not exclude non CHC influences. Additionally, the relatively high proportion of non‐conspecific choices likely reflects baseline orientation errors, which cannot be disentangled without additional negative controls. Consequently, future studies using synthetic compounds or purified CHC extracts would be required to establish causality compound‐specific mechanisms. Sexual dimorphism in insect CHC profiles is widespread and arises from diverse combinations of divergent natural and sexual selection pressures (Wurdack et al. 2015; Moris et al. 2023). While male‐driven selection dominates in these wasps, female choice for male CHCs occurs in other systems, underscoring the pronounced taxon‐specific dynamics (Dyer et al. 2014). Notably, our fig wasps exhibited pronounced intraspecific qualitative/quantitative divergence. This pattern departs from earlier assumptions that interspecific CHC differences are qualitative and intraspecific ones quantitative (Martin et al. 2008). We speculate that this pattern could be associated with two potential factors: (1) Sex‐specific life history strategies post‐emergence: Females navigate external environments (variable humidity/temperature) for host‐fig location, may benefit from elevated CHC quantities for desiccation resistance and signaling. Males, confined to enclosed fig cavities, may rely on mate recognition with simpler CHC profiles. (2) Haplodiploidy constraints: Males, being haploid, inherit only maternal alleles, may constrain CHC diversity compared to diploid females. This genetic asymmetry potentially contribute intersexual divergence, as seen in other haplodiploid systems (Blomquist and Ginzel 2021). Therefore, the relative contributions of genetic architecture versus mating systems to CHC divergence merit further investigation.

Among the six key compounds identified by random forest analysis, the methylalkane 2‐Me‐C28, shared across all five wasp species and seasonally stable, may represent a candidate sexual pheromone, mirroring methyl‐branched compound roles in other insects (e.g., tsetse flies) (Engl et al. 2018; Simmons et al. 2022). Their identification provides a useful framework for prioritizing compounds in future functional studies.

Intriguingly, female CHC profiles exhibited greater interspecific separation than males (Figure 1), suggesting stronger selection on female chemical specificity. In fig wasps exhibiting pronounced sexual dimorphism (e.g., wingless males that mate within the fig), mating is typically male‐driven: emerging first, males engage in a two‐stage recognition process, initial gall localization via antennal probing followed by contact‐based species confirmation (Ghara and Borges 2010; Krishnan et al. 2014; Liu et al. 2019). Similar mating patterns have also been reported in several non‐pollinating fig wasp lineages (Pereira and Prado 2005; Felício and Pereira 2023). Sex‐specific CHC differentiation evolves when: mating decisions are sex‐biased (here, male‐driven), and fitness costs of hybridization (e.g., sterile offspring) outweigh recognition trade‐offs (Buellesbach et al. 2013). The observed patterns thus support CHCs as critical signals for male‐mediated species recognition, reducing cross‐species mating in sympatry.

### 4.2 Cuticular Hydrocarbon Profiles Reveals Potential Functional Group Differentiation Within a Host–Community System

Niche overlap and competitive divergence analyses demonstrated that sympatric species sharing ecological resources develop convergent traits (Pastore et al. 2021), as evidenced by hierarchical clustering of rainy‐season CHC profiles in five *F. semicordata*‐associated fig wasps. Although our sampling encompassed only a limited number of species per functional guild‐reflecting the restricted fig wasp community within *F. semicordata* syconia‐females within equivalent functional guilds (gall‐makers, kleptoparasites, parasitoids) showed strong CHC clustering, supporting an ecological association between CHC composition and functional differentiation within this host–community system. This pattern reflects chemical trait associations observed within a single host fig context and provides an empirical example for exploring functional structuring in fig wasp communities. Future studies incorporating expanded taxonomic sampling across diverse *Ficus* mutualisms are essential to assess the generality of these patterns. As diet composition was not directly measured in this study, we propose the following as a possible hypothesis: host‐mediated dietary differences likely underpin guild‐specific CHC signatures: gall‐makers (e.g., *C. gravelyi* , *P. dunia*) consume seed endosperm or galled ovaries, while kleptoparasites (*S. cunia*) exploit pre‐formed galls, and parasitoids (*Apocrypta* sp., *S. trifemmensis*) target larvae and plant tissues (Compton and Hawkins 1992; Weiblen and Bush 2002). Such divergence in resource use may be associated with the observed divergent CHC profiles, as evidenced by the axis 1 of the NMDS ordination clearly separating phytophagous species (gall‐makers and kleptoparasites) from parasitoid species. Similarly, in other insect systems, has been shown to influence chemical signaling traits and to contribute to mate preference, as documented in beetles (e.g., *Phaedon cochleariae*) (Geiselhardt et al. 2012). Although direct experimental evidence for this mechanism is currently lacking in fig wasps, it provides a plausible ecological framework for interpreting CHCs differentiation among functional groups. Ecological diversification into functional guilds reflects adaptive resource partitioning within the fig‐wasp mutualism (Kerdelhué and Rasplus 1996). Against this backdrop, CHCs, while firmly established as contact pheromones mediating male mate recognition, are also hypothesized to function as critical chemical cues enabling females to identify suitable oviposition sites (Elias et al. 2018; Yadav et al. 2018). Non‐pollinating females, tasked with locating oviposition sites, could use CHCs to avoid exploited galls (under the “one wasp per flower” constraint) or to detect competitors. Parasitoids might exploit these cues to locate hosts, enhancing reproductive success (Weiblen 2002; Yadav et al. 2018). This functional differentiation explains why guild‐specific CHC patterns were more pronounced in females; males, uninvolved in oviposition, lack selective pressure for such chemical specialization.

Interspecific competition, a key driver of mutualistic community structure (Palmer et al. 2003), peaks during rainy seasons when fig wasp abundance and diversity surge (Xie et al., unpublished). Correspondingly, guild‐specific CHC clustering was seasonally restricted to this period, suggesting that CHC composition may respond to seasonal variation in resource availability and interaction intensity (Proffit et al. 2007; Agarwal and Althoff 2024). This plasticity allows dynamic adaptation to fluctuating resource availability, aligning with frameworks where obligate mutualisms evolve specificity through intra‐guild competition (Agarwal and Althoff 2024). Even without direct adult interactions, CHCs may reflect indirect competition shaped by selection for divergent oviposition strategies or avoidance of previously exploited syconia, chemically mediated through compound synergies and antagonisms (Blomquist and Ginzel 2021; Simmons et al. 2022).

### 4.3 CHC Profiles Differentiation Among Functional Ecological Groups Across Seasonal Regimes

CHCs are critical for insect survival under climatic stress, balancing waterproofing and chemical communication (Gibbs and Pomonis 1995; Sprenger et al. 2018; Kárpáti et al. 2023). In five fig wasp species associated with *F. semicordata*, we observed distinct species‐specific plasticity in CHC composition in response to variations in temperature and precipitation, suggesting that climatic conditions may shape their chemical profiles through distinct pathways. Under dry‐hot conditions (high heat/low rainfall), gall‐makers ( *C. gravelyi* ) and kleptoparasites (*S. cunia*) increased long‐chain n‐alkanes (e.g., n‐C34, n‐C36, n‐C40, and n‐C44) while reducing methyl‐branched alkanes (2‐Me‐C28) and methylalkenes (squalene). This aligns with desiccation‐resistance strategies in ants (*Myrmica* spp.) (Sprenger et al. 2018) and wasps (*Polistes* spp.) (Michelutti et al. 2018), where linear alkanes enhance waterproofing. Conversely, parasitoids exhibited divergent responses: *S. trifemmensis* elevated methyl‐branched compounds, while *Apocrypta* sp. increased alkenes and methylalkenes during dry periods. These differences indicate that even under the same host and climatic background, different species may employ distinct chemical regulatory strategies to cope with environmental stress. Notably, seasonal CHC variation may also reflect fig microenvironmental conditions or host plant chemistry, as fig wasps develop inside syconia and are not directly exposed to ambient climate.

The consistent decline of 2‐Me‐C28 across all species under seasonally drier conditions, combined with its strong contribution to sex‐ and species‐level discrimination in random forest analyses, raises the possibility that this compound may be more closely associated with chemical recognition or signaling than with waterproofing. Future behavioral or electrophysiological studies could help test this hypothesis and further clarify whether 2‐Me‐C28 serves a primarily signaling or structural/physiological function.

Pollinators exhibited significantly higher total CHC content than non‐pollinators, particularly in dry seasons. This likely reflects ecological niche differences: pollinators disperse via wind‐exposed canopies, necessitating robust waterproofing, while non‐pollinators occupy shaded understories with reduced desiccation pressure (Compton et al. 2000; Harrison and Rasplus 2006; Ahmed et al. 2009). Previous studies indicate that the waterproofing efficiency of CHCs depends more on hydrocarbon structure than on bulk quantity (Gefen et al. 2015), suggesting that compositional restructuring, rather than simple accumulation, may represent an important mechanism for coping with prolonged aridity. In this context, a notable increase in the specific alkene C29:1 was detected uniquely in *S. trifemmensis* during dry periods, reaching > 40% of total CHCs. This finding highlights the unique plasticity of its CHC composition in response to climatic changes. The functional significance of alkene biosynthesis in this species remains unknown. However, given that n‐alkanes are generally considered more effective for desiccation resistance, the observed prioritization of alkenes is unexpected. We propose two hypotheses: (1) alkenes may enhance cuticular pliability, reducing heat‐induced structural damage; and (2) Parasitoids might allocate limited resources to competing traits (e.g., immune defense or chemical signaling) at the expense of optimal waterproofing. The inverse relationship between alkene and n‐alkane levels across guilds, *C. gravelyi* /*S. cunia* (n‐alkane‐dominated) versus *S. trifemmensis* (alkene‐rich), suggests a potential trade‐off or linkage in metabolic allocation between these compound classes (Menzel et al. 2017).

### 4.4 Sex‐Specific Adaptive Divergence Across Seasonal Regimes

Intraspecific trait variation underpins adaptive responses to environmental pressures, with multifunctional traits like CHCs balancing survival and reproductive demands (Zhang et al. 2008; Dyer et al. 2014; Chung and Carroll 2015; Li et al. 2024). Under dry‐hot conditions (high temperatures and/or low precipitation), females of *C. gravelyi* and *S. cunia* exhibited increased proportions of long‐chain n‐alkanes (C34, C36, C40, C44) and decreased methyl‐branched alkanes (2‐Me‐C28) and methylalkenes (e.g., squalene), whereas males displayed inverse patterns with reduced n‐alkanes and elevated alkenes/methylalkenes. These sex‐specific CHC shifts may reflect differential selection pressures from chemical signaling imperatives, waterproofing demands, and physiological trade‐offs (Blows 2002; Berson Jacob et al. 2019). Seasonal NMDS ordination aligned CHC profiles with morphological traits (Figures S7 and S9): fog‐cool and rainy seasons clustered closely, while dry‐hot profiles diverged. This parallels *Trypoxylus dichotomus* beetles, where CHCs encode body size information (Bell et al. 2024). CHC plasticity correlates with ecological niche partitioning (Otte et al. 2018; Li et al. 2024). We observed opposing seasonal CHC adaptations between gall‐makers/kleptoparasites ( *C. gravelyi* /*S. cunia*) and parasitoids (*Apocrypta* sp., *S. trifemmensis*), a divergence that likely reflects differential metabolic constraints across trophic levels. A striking sex‐linked pattern emerged: females of four species (excluding *Apocrypta* sp.) exhibited significantly lower squalene proportions than males (*p* < 0.01). Squalene, astructurally complex methylalkene with six methyl groups and double bonds, may serve dual roles in males (Kather and Martin 2015; Blomquist and Ginzel 2021). Its oxygen‐binding capacity, documented in moles (Downing and Stewart 1987), could enhance hypoxia tolerance within the fig's O_2_‐depleted microhabitat, benefiting early‐emerging males. Elevated male squalene under seasonally drier conditions further hints at desiccation‐resistance roles, though its rarity in arthropods demands functional validation.

In summary, fig wasps associated with *F. semicordata* exhibit stable yet plastic in CHCs across species, functional groups, and sexes. Species‐specific CHC profiles with sexual dimorphism, combined with male mate choice, constitute a plausible chemical basis for mate recognition and may contribute to reducing signal overlap in sympatric assemblages. Functional groups sharing resource‐acquisition strategies exhibit parallel CHC patterns, whereas co‐occurring species maintain chemical distinctiveness under shared host and climatic conditions through adjustments in compound composition and relative abundance. Future work integrating sensory perception and functional validation across broader taxonomic contexts will be critical for evaluating the generality and mechanistic basis of this process.

## Author Contributions

**Hua Xie:** data curation (equal), formal analysis (equal), investigation (equal), software (equal), supervision (equal), validation (equal), visualization (equal), writing – original draft (lead). **Shouxian Zhang:** data curation (equal), formal analysis (equal), investigation (equal), software (equal), writing – review and editing (supporting). **Yonghui Zhu:** data curation (equal), formal analysis (equal), investigation (equal), software (equal), writing – review and editing (supporting). **Subo Shao:** data curation (supporting), formal analysis (supporting), investigation (supporting), visualization (equal), writing – review and editing (supporting). **Pei Yang:** conceptualization (equal), funding acquisition (equal), project administration (equal), writing – review and editing (equal). **Zongbo Li:** conceptualization (equal), funding acquisition (equal), project administration (equal), resources (equal), writing – original draft (equal), writing – review and editing (equal). **Yuan Zhang:** conceptualization (equal), funding acquisition (equal), project administration (equal), resources (equal), writing – original draft (equal), writing – review and editing (equal).

## Funding

This work was supported by Yunnan Fundamental Research Projects, 202401AT070265, 202401BD070001‐111. The First Class Forestry Academic Subject in Yunnan Province, 523003. The Young Top‐Notch Talent of Yunnan Outstanding Talent Program, YNWR‐QNBJ‐2018‐131; YNWR‐QNBJ‐2019‐123; XDYC‐QNRC. National Natural Science Foundation of China, 32260719, 32160296, 31760107.

## Conflicts of Interest

The authors declare no conflicts of interest.

## Supporting information


**Table S1:** Number of biological replicates for male and female fig wasps associated with *Ficus semicordata* across seasons.
**Table S2:** Treatments and replicates in male mate choice experiments.
**Table S3:** Absolute mean content (mean ± SE, ng/unit) and critical characterization of sex‐specific cuticular hydrocarbons in fig wasps (male and female) associated with *Ficus semicordata*. Please note that key contributing compounds (bold) were selected from the top‐ranked variables (Mean Decrease Gini), with the optimal number of variables (*n* = 6) determined by 10‐fold cross‐validation (five repeats) based on minimum error.
**Table S4:** Permutational multivariate ANOVA (PERMANOVA) was performed to assess the dissimilarities in community structure among fig wasp assemblages associated with *Ficus semicordata*.
**Table S5:** Sex‐specific absolute quantities of cuticular hydrocarbon profiles in four dominant fig wasp species associated with *Ficus semicordata* were analyzed across three distinct seasons.
**Table S6:** Permutational multivariate ANOVA (PERMANOVA) was conducted to evaluate the multivariate responses of distinct compound classes to environmental factors. Statistically significant *p*‐values (*p* < 0.05) are indicated in bold.
**Figure S1:** Female oviposition sequences and behaviors across syconium developmental phases in *Ficus semicordata*. Solid lines denote the first 50% of wasps arriving at the syconium, while dashed lines represent subsequent arrivals. Capital letters (A–E) above Latin species names correspond to the five fig wasp species depicted in the left/right panels, illustrating their oviposition activity within the syconium.
**Figure S2:** Cross‐validation error as a function of the number of variables in the random forest model. The optimal number of variables (*n* = 6) was selected based on the minimum error.
**Figure S3:** Non‐metric multidimensional scaling (NMDS) ordination of cuticular hydrocarbon profiles in dominant fig wasp species associated with *Ficus semicordata* during the dry‐hot season.
**Figure S4:** Non‐metric multidimensional scaling (NMDS) ordination of cuticular hydrocarbon profiles in dominant fig wasp species associated with *Ficus semicordata* during the fog‐cool season.
**Figure S5:** Hierarchical clustering analysis of cuticular hydrocarbon profiles in females (A) and males (B) of four dominant fig wasp species during the dry‐hot season: *Ceratosolen gravelyi* (Cgra), *Sycophaga cunia* (Scun), *Apocrypta* sp. (Apoc), and *Sycoscapter trifemmensis* (Stri).
**Figure S6:** Hierarchical clustering analysis of cuticular hydrocarbon profiles in females (A) and males (B) of four dominant fig wasp species during the fog‐cool season: *Ceratosolen gravelyi* (Cgra), *Sycophaga cunia* (Scun), *Apocrypta* sp. (Apoc), and *Sycoscapter trifemmensis* (Stri).
**Figure S7:** Non‐metric multidimensional scaling (NMDS) ordination of cuticular hydrocarbon profiles in dominant fig wasp species associated with *Ficus semicordata* across seasonal variations.
**Figure S8:** Hierarchical clustering analysis of cuticular hydrocarbon (CHC) profiles in females (A) and males (B) across all samples pooled from four dominant fig wasp species, with samples colored by season (fog–cool, rainy, and dry–hot): *Ceratosolen gravelyi* (Cgra), *Sycophaga cunia* (Scun), *Apocrypta* sp. (Apoc), and *Sycoscapter trifemmensis* (Stri).
**Figure S9:** Principal Component Analysis (PCA) of morphological variation in dominant fig wasp species associated with *Ficus semicordata* across seasonal shifts.

## Data Availability

The data and code used in this study are openly available in figshare at https://doi.org/10.6084/m9.figshare.29261480.v1.
