# Supplementary material for: Cuticular Hydrocarbons‐Revealed Functional Groups and Seasonal Acclimation in Sympatric Fig Wasp Mating Assemblages
Source: Ecol Evol. 2026 Jul 7;16(7):e73976. doi: 10.1002/ece3.73976 (PMC13341148; doi:10.1002/ece3.73976)
Supplement: Supplementary file 1 — Table S1: Number of biological replicates for male and female fig wasps associated with Ficus semicordata across seasons. Table S2: Treatments and replicates in male mate choice experiments. Table S3: Absolute mean content (mean ± SE, ng/unit) and critical characterization of sex‐specific cuticular hydrocarbons in fig wasps (male and female) associated with Ficus semicordata. Please note that key contributing compounds (bold) were selected from the top‐ranked variables (Mean Decrease Gini), with the optimal number of variables (n = 6) determined by 10‐fold cross‐validation (five repeats) based on minimum error. Table S4: Permutational multivariate ANOVA (PERMANOVA) was performed to assess the dissimilarities in community structure among fig wasp assemblages associated with Ficus semicordata. Table S5: Sex‐specific absolute quantities of cuticular hydrocarbon profiles in four dominant fig wasp species associated with Ficus semicordata were analyzed across three distinct seasons. Table S6: Permutational multivariate ANOVA (PERMANOVA) was conducted to evaluate the multivariate responses of distinct compound classes to environmental factors. Statistically significant p‐values (p < 0.05) are indicated in bold. Figure S1: Female oviposition sequences and behaviors across syconium developmental phases in Ficus semicordata. Solid lines denote the first 50% of wasps arriving at the syconium, while dashed lines represent subsequent arrivals. Capital letters (A–E) above Latin species names correspond to the five fig wasp species depicted in the left/right panels, illustrating their oviposition activity within the syconium. Figure S2: Cross‐validation error as a function of the number of variables in the random forest model. The optimal number of variables (n = 6) was selected based on the minimum error. Figure S3: Non‐metric multidimensional scaling (NMDS) ordination of cuticular hydrocarbon profiles in dominant fig wasp species associated with Ficus semicordata during the dry [file ECE3-16-e73976-s001.docx]

**Supplementary Methods**

*Interspecific vs. intraspecific differences*

NMDS was performed using the metaMDS() function from the vegan package with Bray-Curtis dissimilarity matrices. Prior to analysis, CHC data underwent square-root transformation and Wisconsin double standardization to reduce dominance effects of abundant compounds. The NMDS was run with autotransform = TRUE (default) and k = 2 dimensions. Stress values were used to quantify model fit (stress < 0.1 considered good).

PERMANOVA was performed using the adonis2() function from the vegan package with 999 permutations, type = “III” sums of squares, and Bray-Curtis distances. To test for significant differences between each pair of species, we conducted pairwise PERMANOVA by subsetting the data to each species pair and recalculating distances to avoid distance matrix distortion due to reduced sample sizes. P-values from pairwise comparisons were adjusted using the FDR correction.

Random forest and recursive feature elimination. Random forest models were implemented using the randomForest package with ntree = 1000 and mtry = 2. Variable importance was assessed based on mean decrease in Gini index. To identify key hydrocarbons driving species differentiation, we performed recursive feature elimination (RFE) using the rfcv() function with 10-fold cross-validation (5 repeats). The step = 1.5 setting resulted in approximately one-third of variables being removed at each iteration. The optimal feature set was determined by identifying the inflection point of the cross-validation error curve. Final variable importance was ranked by mean decrease in Gini index.

Hierarchical clustering was performed using the unweighted pair-group method with arithmetic mean (UPGMA) based on Bray-Curtis distance matrices. The Bray-Curtis dissimilarity matrix was calculated using the vegdist() function from the vegan package with method = 'bray'. UPGMA clustering was implemented using the hclust() function with method = 'average'.

*Seasonal variation*

Seasonal differences in CHC compound abundances were assessed using non-parametric methods due to non-normal distributions (Shapiro-Wilk test, P < 0.05). For each species and hydrocarbon class, the Kruskal-Wallis rank sum test (kruskal.test() in base R) was first applied to evaluate overall differences among seasons (rainy, dry-hot, fog-cool). Where significant differences were detected (P < 0.05), post-hoc pairwise comparisons were performed using the Nemenyi test implemented in the PMCMRplus package. The Nemenyi test is a non-parametric multiple comparison procedure based on rank sums, providing adjusted P-values for all pairwise seasonal comparisons.

*Climate effects*

To test the effects of climate on CHC composition, we performed separate PERMANOVA models (999 permutations, Euclidean distances) for each of the four hydrocarbon classes (n-alkanes, methylalkanes, alkenes, and methylalkenes) and for the total absolute content of CHCs, using the adonis2() function from the vegan package.

For each model, the dissimilarity matrix of the respective hydrocarbon class was used as the response variable, with seasonal mean temperature (°C) and cumulative precipitation (mm) as predictors. Type III sums of squares were used (default in adonis2), meaning each predictor was evaluated conditional on the other.

Prior to analysis, compound abundances within each hydrocarbon class were standardized using the decostand() function with method = 'standardize' (z-score transformation: mean = 0, standard deviation = 1). Euclidean distance matrices were then calculated using the vegdist() function with method = 'euclidean'.

Multiple comparison correction. As separate PERMANOVA models were performed for each hydrocarbon class across species and sexes, we applied FDR correction (Benjamini-Hochberg, 1995) to control for false discovery rates. Adjusted P-values < 0.05 were considered statistically significant.

Climate data were obtained from the China Meteorological Data Network (http://data.cma.cn). Mean seasonal air temperature (°C) and cumulative seasonal precipitation (mm) were calculated for the sampling period (June 2020 – May 2021). Climate variables were matched to each sample according to its collection season, defined as rainy (June–October), dry-hot (March–May), and fog-cool (November–February).

*Morphological analysis*

Morphological variation was explored using principal component analysis (PCA) based on five morphometric traits: body length, head length, head width, hind tibia length, and ovipositor length. Prior to analysis, all variables were standardized using z-scores (scale.unit = TRUE), ensuring that the PCA was based on a correlation matrix to avoid dominance by traits with larger absolute values. In the PCA ordination, individuals were labeled and colored by species, shaped by sex, and visually distinguished by season to allow qualitative assessment of seasonal patterns in morphospace. While PCA is an exploratory method and does not test formal hypotheses, we used it to qualitatively evaluate whether seasonal clustering in morphological traits paralleled the patterns observed in CHC composition.

*Behavioral assays*

Male orientation responses toward female CHCs were assessed using Fisher’s exact test (excluding non-responding individuals). For repeated binary responses (coded as 0/1) across experimental trials, Cochran’s Q test evaluated preference consistency across repeated trials, with significant outcomes followed by McNemar’s test (FDR-adjusted P-values).

**Legend**

**Fig.S1** Female oviposition sequences and behaviors across syconium developmental phases in *Ficus semicordata*. Solid lines denote the first 50% of wasps arriving at the syconium, while dashed lines represent subsequent arrivals. Capital letters (A–E) above Latin species names correspond to the five fig wasp species depicted in the left/right panels, illustrating their oviposition activity within the syconium.

**Fig.S2** Cross-validation error as a function of the number of variables in the random forest model. The optimal number of variables (n = 6) was selected based on the minimum error.

**Fig.S3** Non-metric multidimensional scaling (NMDS) ordination of cuticular hydrocarbon profiles in dominant fig wasp species associated with *Ficus semicordata* during the dry-hot season.

**Fig.S4** Non-metric multidimensional scaling (NMDS) ordination of cuticular hydrocarbon profiles in dominant fig wasp species associated with *Ficus semicordata* during the fog-cool season.

**Fig.S5** Hierarchical clustering analysis of cuticular hydrocarbon profiles in females (A) and males (B) of four dominant fig wasp species during the dry-hot season: *Ceratosolen gravelyi* (Cgra), *Sycophaga cunia* (Scun), *Apocrypta* sp. (Apoc), and *Sycoscapter trifemmensis* (Stri).

**Fig.S6** Hierarchical clustering analysis of cuticular hydrocarbon profiles in females (A) and males (B) of four dominant fig wasp species during the fog-cool season: *Ceratosolen gravelyi* (Cgra), *Sycophaga cunia* (Scun), *Apocrypta* sp. (Apoc), and *Sycoscapter trifemmensis* (Stri).

**Fig.S7** Non-metric multidimensional scaling (NMDS) ordination of cuticular hydrocarbon profiles in dominant fig wasp species associated with *Ficus semicordata* across seasonal variations.

**Fig.S8** Hierarchical clustering analysis of cuticular hydrocarbon (CHC) profiles in females (A) and males (B) across all samples pooled from four dominant fig wasp species, with samples colored by season (fog–cool, rainy, and dry–hot): *Ceratosolen gravelyi* (Cgra), *Sycophaga cunia* (Scun), *Apocrypta* sp. (Apoc), and *Sycoscapter trifemmensis* (Stri).

**Fig.S9** Principal Component Analysis (PCA) of morphological variation in dominant fig wasp species associated with *Ficus semicordata* across seasonal shifts.


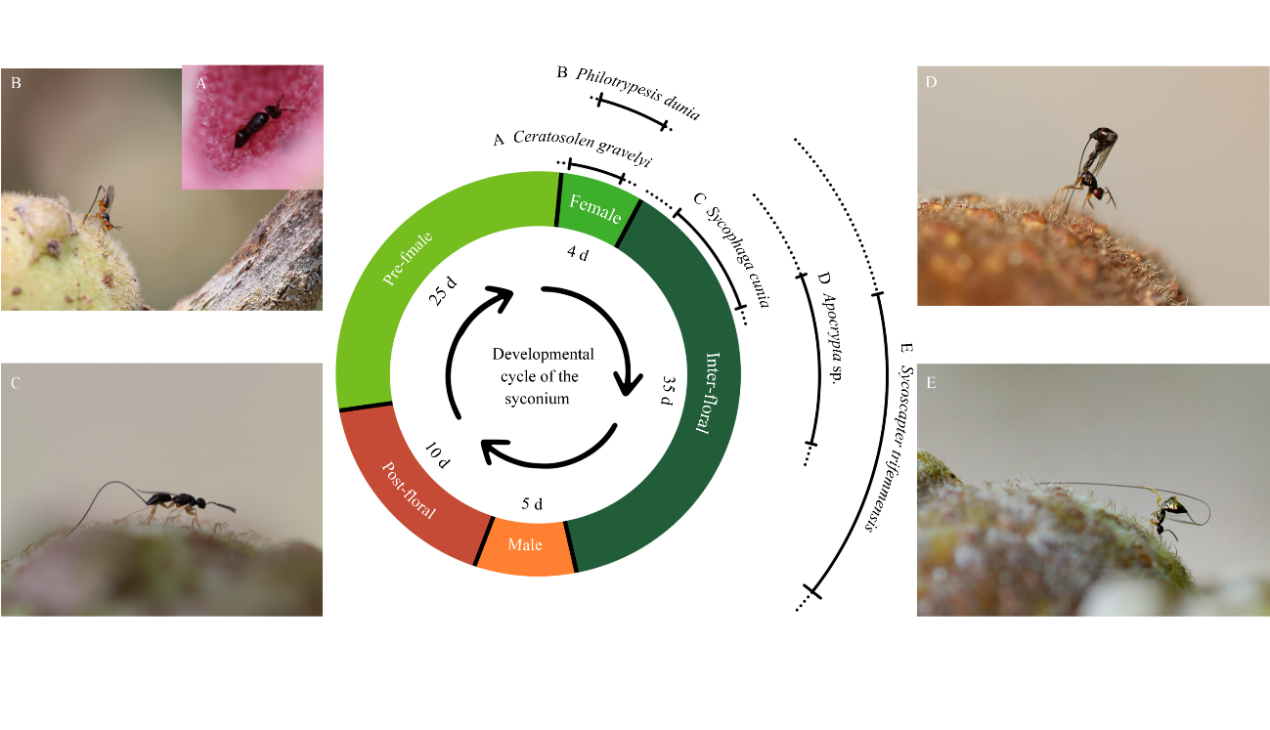


**Fig.S1** Female oviposition sequences and behaviors across syconium developmental phases in *Ficus semicordata*. Solid lines denote the first 50% of wasps arriving at the syconium, while dashed lines represent subsequent arrivals. Capital letters (A–E) above Latin species names correspond to the five fig wasp species depicted in the left/right panels, illustrating their oviposition activity within the syconium.

**
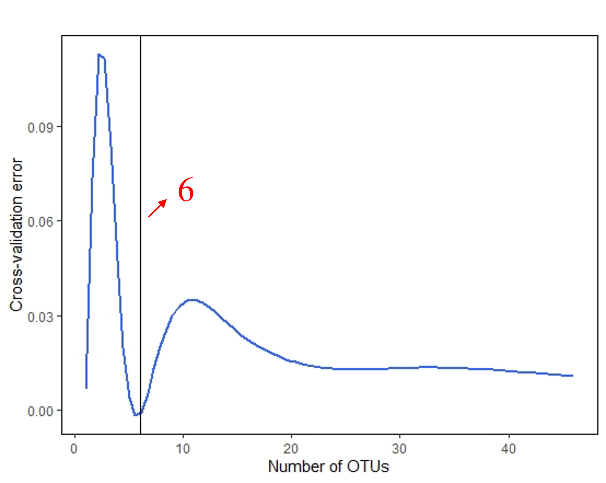
**

**Fig.S2** Cross-validation error as a function of the number of variables in the random forest model. The optimal number of variables (n = 6) was selected based on the minimum error.


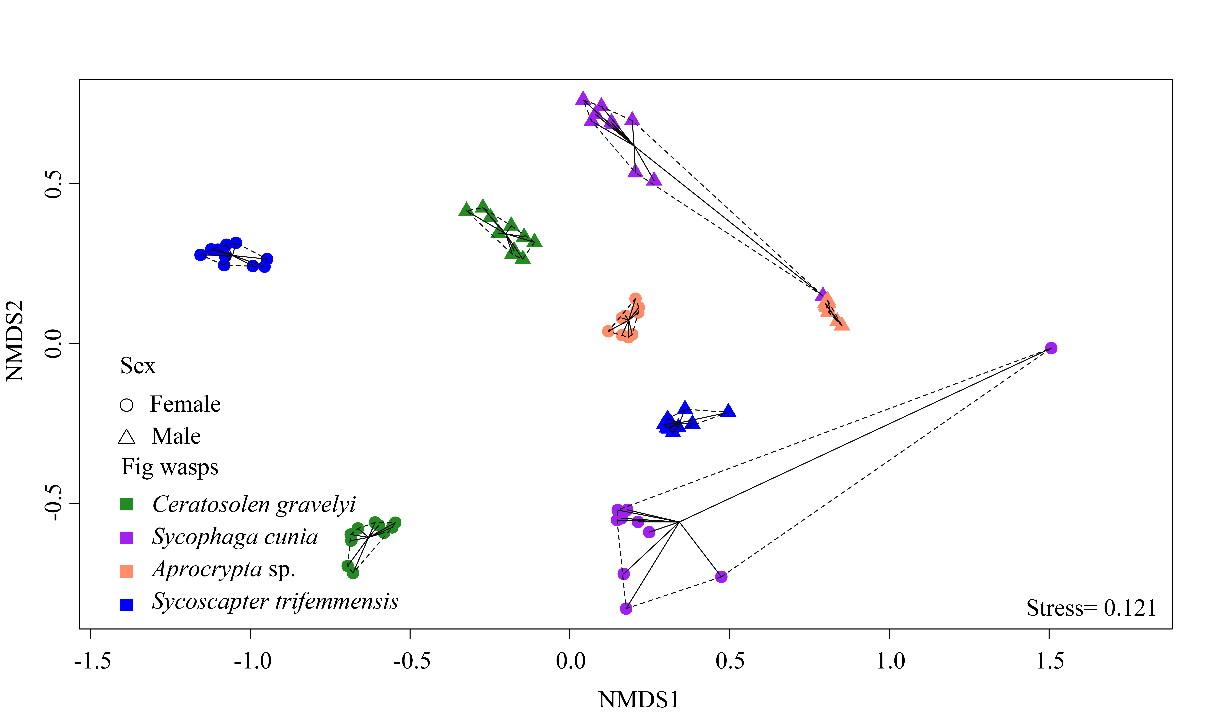


**Fig.S3** Non-metric multidimensional scaling (NMDS) ordination of cuticular hydrocarbon profiles in dominant fig wasp species associated with *Ficus semicordata* during the dry-hot season.


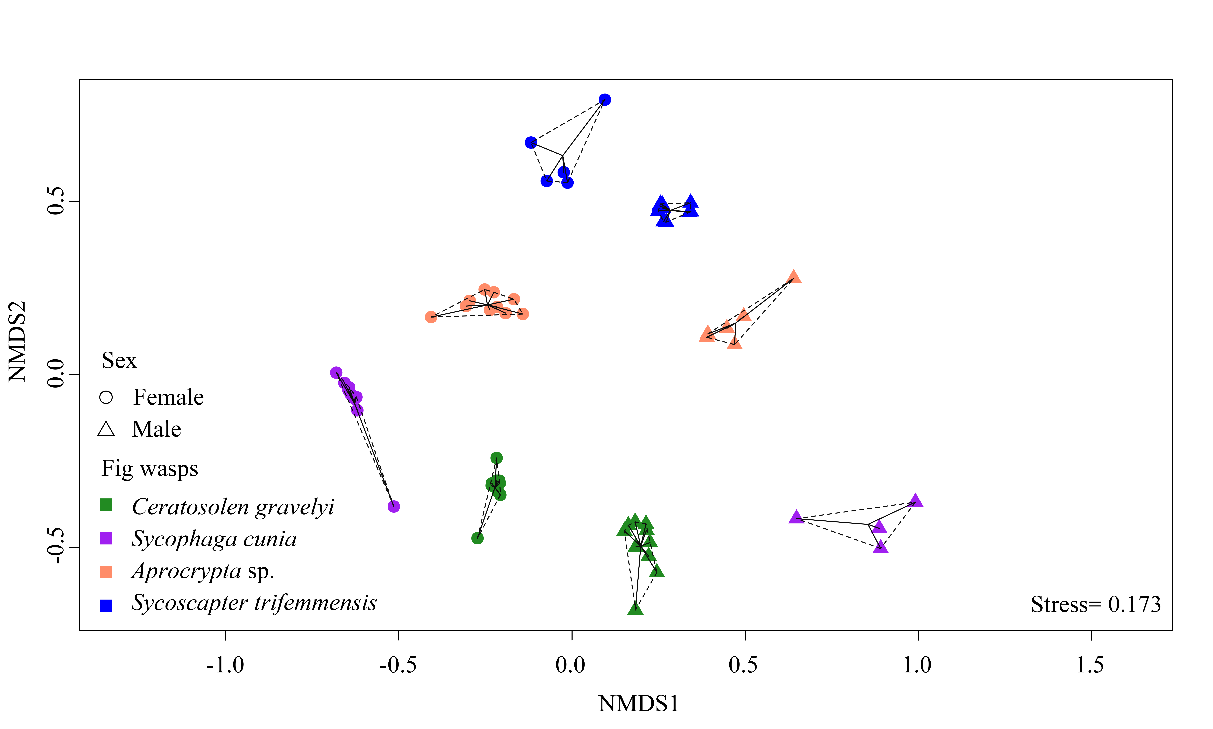


**Fig.S4** Non-metric multidimensional scaling (NMDS) ordination of cuticular hydrocarbon profiles in dominant fig wasp species associated with *Ficus semicordata* during the fog-cool season.

**
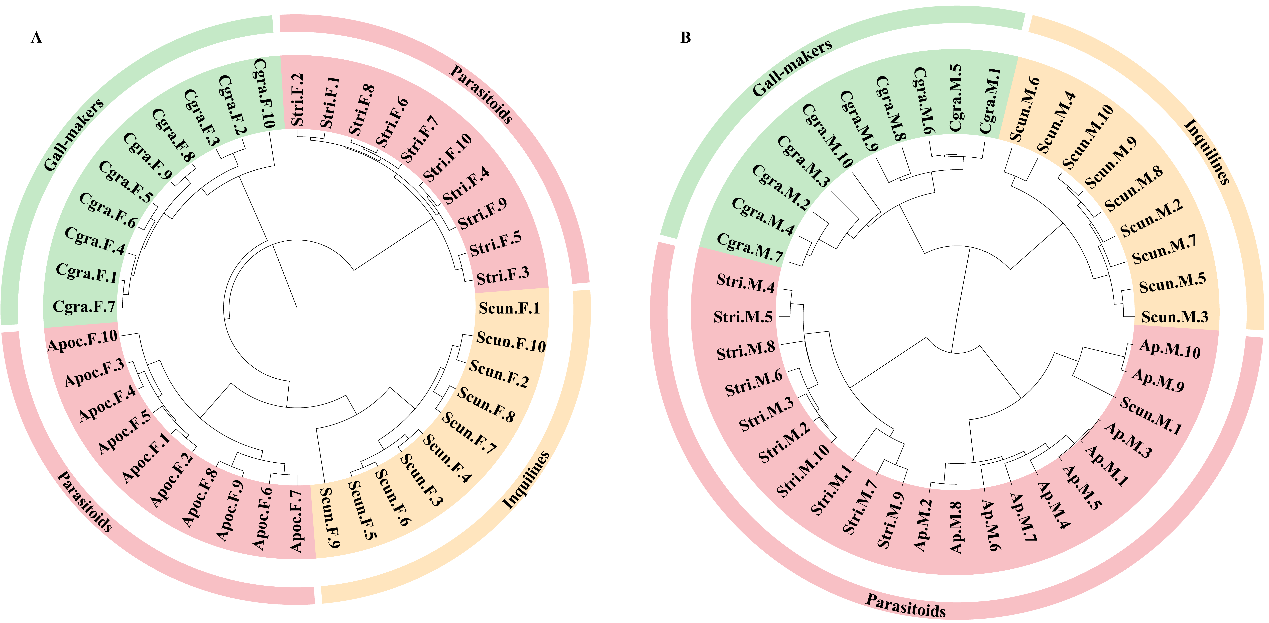
**

**Fig.S5** Hierarchical clustering analysis of cuticular hydrocarbon profiles in females (A) and males (B) of four dominant fig wasp species during the dry-hot season: *Ceratosolen gravelyi* (Cgra), *Sycophaga cunia* (Scun), *Apocrypta* sp. (Apoc), and *Sycoscapter trifemmensis* (Stri).

**
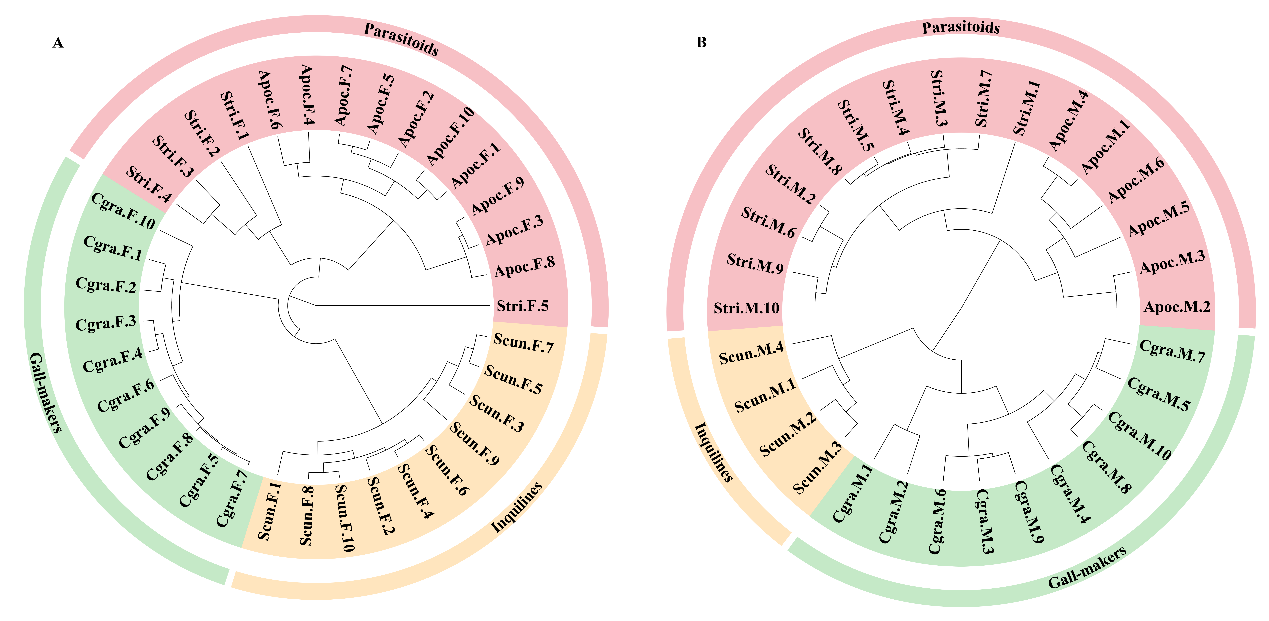
**

**Fig.S6** Hierarchical clustering analysis of cuticular hydrocarbon profiles in females (A) and males (B) of four dominant fig wasp species during the fog-cool season: *Ceratosolen gravelyi* (Cgra), *Sycophaga cunia* (Scun), *Apocrypta* sp. (Apoc), and *Sycoscapter trifemmensis* (Stri).

**
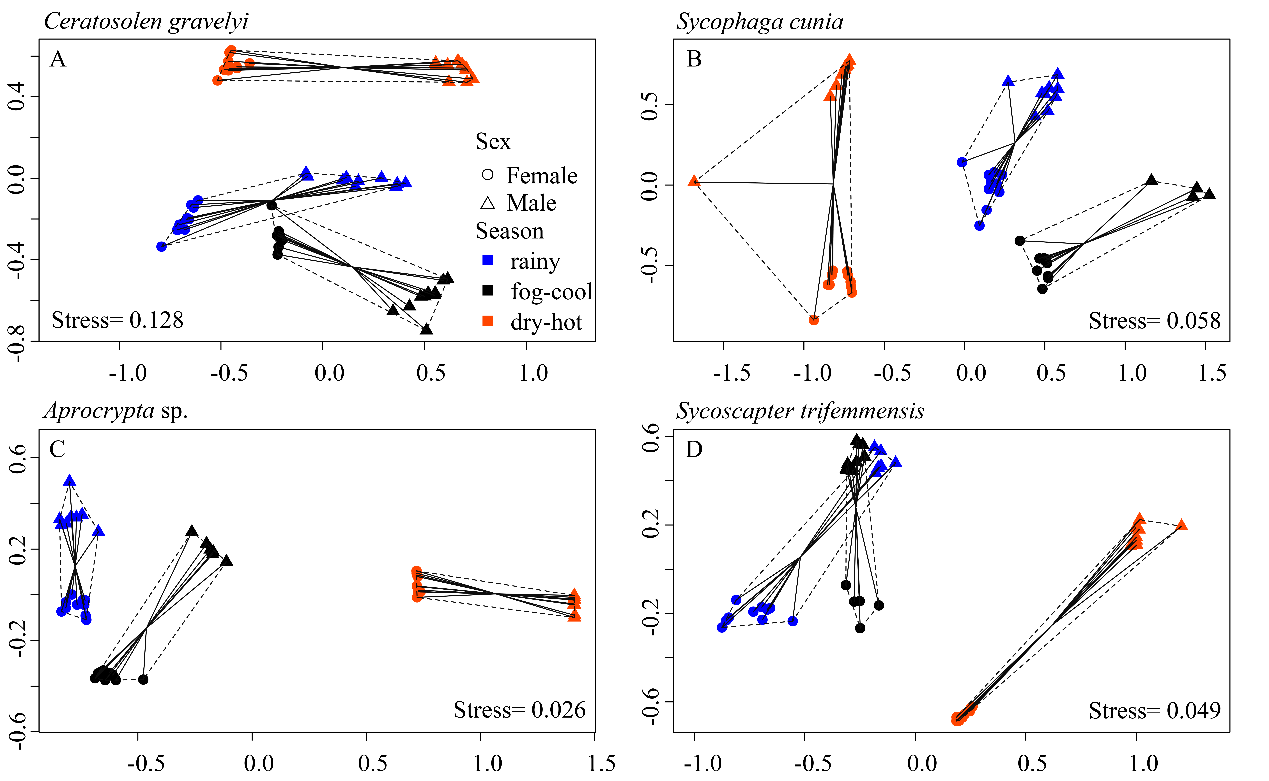
**

**Fig.S7** Non-metric multidimensional scaling (NMDS) ordination of cuticular hydrocarbon profiles in dominant fig wasp species associated with *Ficus semicordata* across seasonal variations.

**
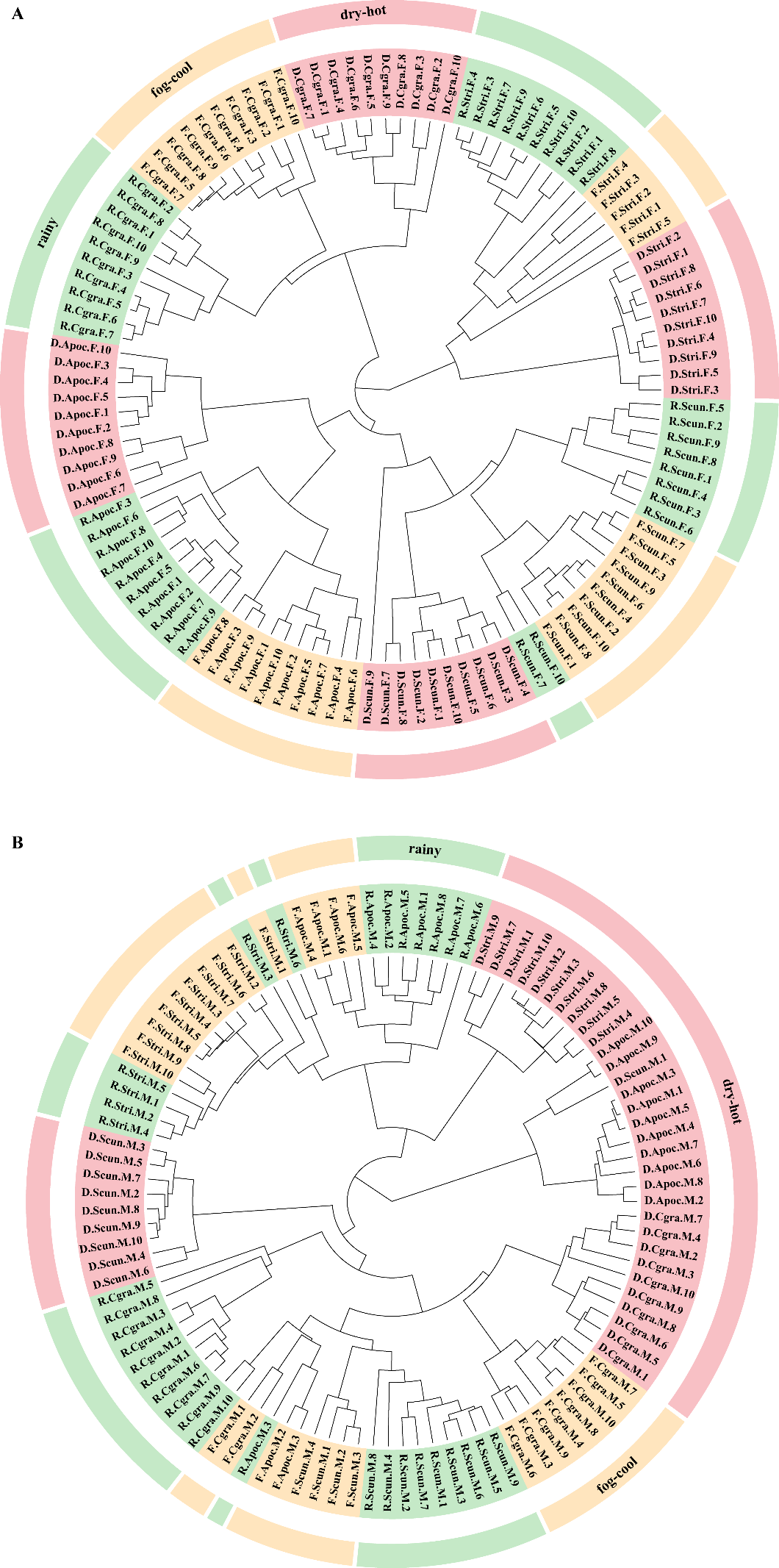
**

**Fig.S8** Hierarchical clustering analysis of cuticular hydrocarbon (CHC) profiles in females (A) and males (B) across all samples pooled from four dominant fig wasp species, with samples colored by season (fog–cool, rainy, and dry–hot): *Ceratosolen gravelyi* (Cgra), *Sycophaga cunia* (Scun), *Apocrypta* sp. (Apoc), and *Sycoscapter trifemmensis* (Stri).

**
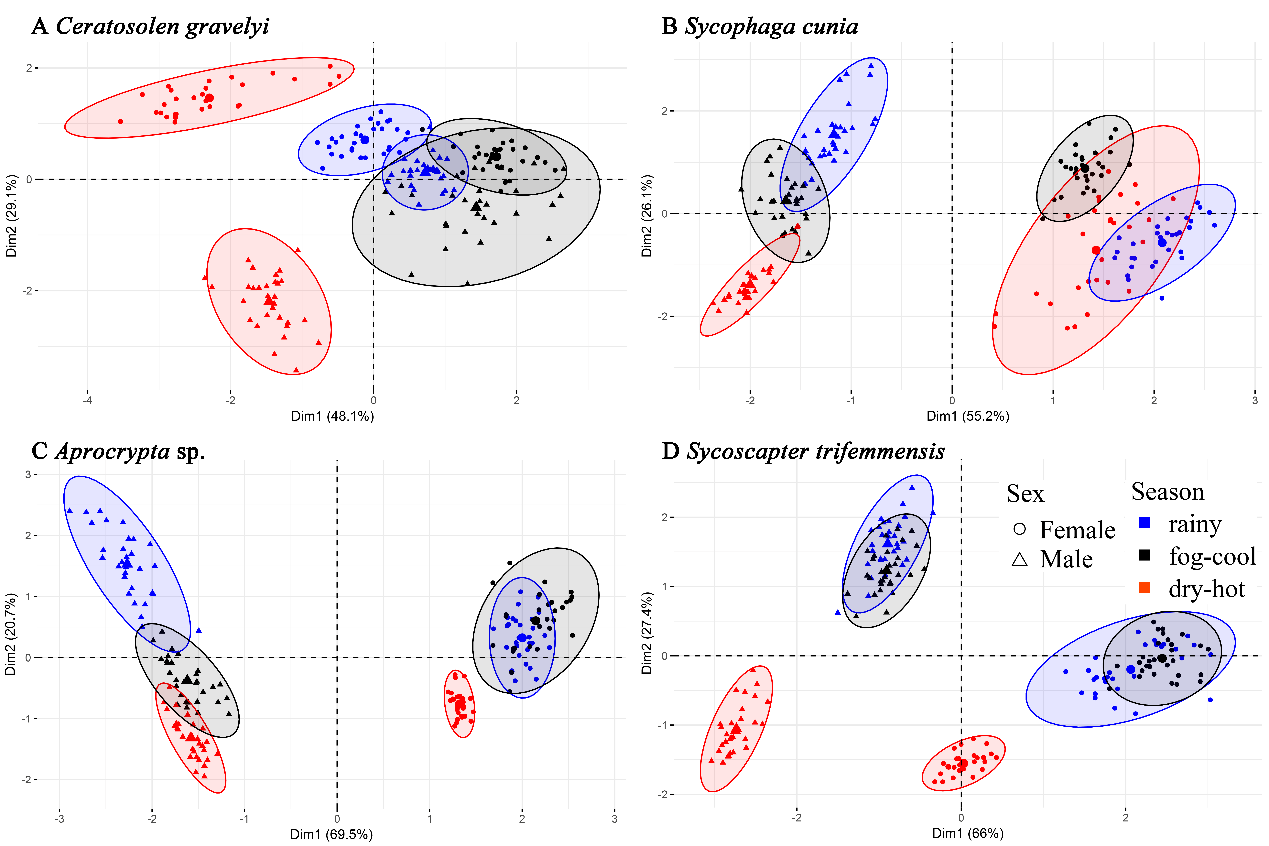
**

**Fig.S9** Principal Component Analysis (PCA) of morphological variation in dominant fig wasp species associated with *Ficus semicordata* across seasonal shifts.

Supplementary Legend

**Table S1** Number of biological replicates for male and female fig wasps associated with *Ficus semicordata* across seasons.

**Table S2** Treatments and replicates in male mate choice experiments.

**Table S3** Absolute mean content (mean ± SE, ng/unit) and critical characterization of sex-specific cuticular hydrocarbons in fig wasps (male and female) associated with *Ficus semicordata*. Please note that key contributing compounds (bold) were selected from the top-ranked variables (Mean Decrease Gini), with the optimal number of variables (n = 6) determined by 10-fold cross-validation (five repeats) based on minimum error.**Table S4** Permutational multivariate ANOVA (PERMANOVA) was performed to assess the dissimilarities in community structure among fig wasp assemblages associated with *Ficus semicordata*.

**Table S5** Sex-specific absolute quantities of cuticular hydrocarbon profiles in four dominant fig wasp species associated with *Ficus semicordata* were analyzed across three distinct seasons.

**Table S6** Permutational multivariate ANOVA (PERMANOVA) was conducted to evaluate the multivariate responses of distinct compound classes to environmental factors. Statistically significant P-values (P < 0.05) are indicated in bold.

**Table S1** Number of biological replicates for male and female fig wasps associated with *Ficus semicordata* across seasons.

| **Season** | **Species** | **Replicate number (n)** | | **Sample size (individuals, n)** |
| --- | --- | --- | --- | --- |
|  |  | **Female** | **Male** |  |
| rainy | *Ceratosolen gravelyi* | 10 | 10 | 50 |
|  | *Philotrypesis dunia* | 6 | 5 |  |
|  | *Sycophaga cunia* | 10 | 9 |  |
|  | *Apocrypta* sp. | 10 | 8 |  |
|  | *Sycoscapter trifemmensis* | 10 | 6 |  |
| dry-hot | *Ceratosolen gravelyi* | 10 | 10 |  |
|  | *Sycophaga cunia* | 10 | 10 |  |
|  | *Apocrypta* sp. | 10 | 10 |  |
|  | *Sycoscapter trifemmensis* | 10 | 10 |  |
| fog-cool | *Ceratosolen gravelyi* | 10 | 10 |  |
|  | *Sycophaga cunia* | 10 | 4 |  |
|  | *Apocrypta* sp. | 10 | 6 |  |
|  | *Sycoscapter trifemmensis* | *5* | 10 |  |

**Table S2** Treatments and replicates in male mate choice experiments.

| Male species | Treatment Group | Stimulus 1 | Stimulus 2 | Replicates (n) |
| --- | --- | --- | --- | --- |
| *C. gravelyi* | Conspecific vs. heterospecific (intact) | Intact *C. gravelyi* | Intact *P. dunia* | 46 |
|  |  | Intact *C. gravelyi* | Intact *S. cunia* | 60 |
|  |  | Intact *C. gravelyi* | Intact *Apocrypta* sp. | 65 |
|  |  | Intact *C. gravelyi* | Intact *S. trifemmensis* | 81 |
|  | Conspecific (intact) vs. conspecific (CHC‑removed) | Intact *C. gravelyi* | CHC‑removed *C. gravelyi* | 79 |
| *S. trifemmensis* | Conspecific vs. heterospecific (intact) | Intact *S. trifemmensis* | Intact *C. gravelyi* | 72 |
|  |  | Intact *S. trifemmensis* | Intact *P. dunia* | 64 |
|  |  | Intact *S. trifemmensis* | Intact *S. cunia* | 66 |
|  |  | Intact *S. trifemmensis* | Intact *Apocrypta* sp. | 66 |
|  | Conspecific (intact) vs. conspecific (CHC‑removed) | Intact *S. trifemmensis* | CHC‑removed *S. trifemmensis* | 66 |

**Table S3** Absolute mean content (mean ± SE, ng/unit) and critical characterization of sex-specific cuticular hydrocarbons in fig wasps (male and female) associated with *Ficus semicordata*. Please note that key contributing compounds (bold) were selected from the top-ranked variables (Mean Decrease Gini), with the optimal number of variables (n = 6) determined by 10-fold cross-validation (five repeats) based on minimum error.

| Compounds | R. T. (min) | *Ceratosolen gravelyi* | | *Philotrypesis dunia* | | *Sycophaga cunia* | | *Apocrypta* sp. | | *Sycoscapter trifemmensis* | | M.D. G. |
| --- | --- | --- | --- | --- | --- | --- | --- | --- | --- | --- | --- | --- |
|  |  | ♀ | **♂** | ♀ | **♂** | ♀ | **♂** | ♀ | **♂** | ♀ | **♂** |  |
| n-C13 | 17.136 | - | - | - | - | - | - | 0.16 ± 0.01 | 0.2 ± 0.01 | 0.23 ± 0.03 | 0.2 ± 0.01 | 2.101 |
| 2,6,10-triMe-C12 | 35.556 | - | - | - | - | - | - | 0.07 ± 0.01 | 0.04 ± 0.02 | - | - | - |
| 2-Me-C16-7-ene | 41.57 | - | - | - | - | - | - | - | 0.13 ± 0.08 | - | - | - |
| n-C17 | 42.119 | 0.18 ± 0.03 | - | - | - | - | - | - | - | 0.13 ± 0.05 | - | - |
| 9-C19:1 | 45.342 | - | - | - | - | - | - | - | 0.66 ± 0.29 | - | - | 1.393 |
| 2,6-diMe-C17 | 44.11 | 0.09 ± 0.03 | - | - | - | - | - | - | - | - | - | - |
| n-C19 | 45.751 | 0.08 ± 0.04 | - | - | - | - | - | 0.09 ± 0.03 | - | - | - | - |
| 1,19-C20:2 | 44.402 | - | - | - | 0.43 ± 0.19 | - | 0.22 ± 0.04 | - | - | - | - | 0.840 |
| C20:1 | 49.499 | - | - | - | - | - | 0.13 ± 0.05 | - | - | - | - | - |
| 10-C21:1 | 48.029 | 1.51 ± 0.32 | - | - | - | - | - | - | - | - | - | 1.353 |
| n-C21 | 48.448 | - | - | - | 2.54 ± 0.63 | - | - | - | - | - | - | 1.055 |
| (1E,9E,17Z)-1,9,17-C22:3 | 48.471 | - | 0.37 ± 0.1 | - | - | 0.11 ± 0.04 | - | - | - | - | - | - |
| 1,21-C22:2 | 49.312 | - | - | 17.79 ± 15.27 | - | - | - | - | - | - | - | 0.783 |
| C22:1 | 49.511 | - | - | - | 6.1 ± 4.82 | - | - | - | - | - | - | 0.767 |
| 2-Me-C22 | 50.344 | - | - | - | - | - | - | 0.53 ± 0.09 | 0.59 ± 0.12 | 0.79 ± 0.16 | - | 2.181 |
| 4-Me-C22 | 50.352 | 4.24 ± 0.68 | 7.84 ± 1.48 | 16.55 ± 8.87 | 1.81 ± 0.51 | - | 0.8 ± 0.17 | - | - | - | - | 2.471 |
| n-C23 | 50.402 | - | - | 272.96 ± 89.06 | - | - | - | - | - | - | - | 0.836 |
| C24:1 | 51.7 | - | - | - | - | - | - | - | 0.14 ± 0.07 | - | - | - |
| (6Z,9Z)-6,9-C25:2 | 52.276 | - | - | 55.48 ± 24.49 | - | - | - | - | - | - | - | 0.753 |
| C25:1 | 52.734 | - | - | - | - | - | - | - | 0.4 ± 0.14 | - | - | 0.865 |
| 2-Me-C24 | 52.448 | - | - | - | - | - | - | - | - | 0.52 ± 0.06 | - | 1.129 |
| n-C25 | 52.822 | - | - | - | - | - | 1.32 ± 0.24 | - | - | 0.3 ± 0.04 | - | 1.739 |
| 3-Me-C25 | 53.627 | - | - | - | - | 1.7 ± 0.31 | 0.94 ± 0.16 | - | - | 0.5 ± 0.11 | - | 2.137 |
| C27:1 | 55.005 | - | - | - | - | 2.27 ± 0.43 | 3.53 ± 0.5 | - | - | - | - | 1.616 |
| 2-Me-C26 | 54.807 | - | - | - | - | - | - | 1.09 ± 0.18 | - | 1.75 ± 0.31 | - | 2.096 |
| **n-C27** | 55.728 | - | 2.92 ± 0.53 | - | 1.73 ± 0.37 | - | 1.13 ± 0.12 | 0.55 ± 0.08 | 0.57 ± 0.09 | 2.47 ± 1.5 | - | **2.735** |
| 3-Me-C27 | 56.512 | - | - | - | - | - | - | - | - | 5.07 ± 1.43 | - | 1.250 |
| Squalene | 57.645 | 11.14 ± 1.5 | 62.34 ± 16.01 | 9.73 ± 2.09 | 42.7 ± 10.87 | 16.56 ± 2.8 | 27.02 ± 2.56 | 20.62 ± 1.87 | 7.62 ± 2.29 | - | 1.67 ± 0.43 | 2.406 |
| **2-Me-C28** | 58.124 | 69.67 ± 8.77 | 34.56 ± 7.04 | 39.86 ± 14.82 | 14.35 ± 3.15 | 8.43 ± 1.34 | 5 ± 0.74 | 16.22 ± 1.61 | 3.19 ± 0.78 | 28.92 ± 1.3 | 2.03 ± 0.21 | **2.515** |
| C29:1 | 58.322 | - | 6.07 ± 2.39 | - | 4.39 ± 1.22 | 3.85 ± 0.74 | 4.97 ± 0.64 | 1.55 ± 0.15 | 1.36 ± 0.31 | - | 1.05 ± 0.27 | 1.842 |
| n-C29 | 58.596 | 3.18 ± 0.39 | 9.75 ± 1.95 | 3.6 ± 1.09 | 3.95 ± 0.82 | 1.96 ± 0.46 | 3.46 ± 0.25 | 2.93 ± 0.45 | 1.74 ± 0.14 | 4.59 ± 0.7 | 2.9 ± 0.44 | 1.374 |
| **15-Me-C29** | 58.935 | - | 6.03 ± 1.73 | 9.3 ± 2.13 | - | - | - | 1.72 ± 0.18 | - | 11.34 ± 1.68 | 1.85 ± 0.42 | **2.907** |
| n-C31 | 59.328 | - | - | 13.55 ± 6.14 | - | - | 4.82 ± 0.75 | 3.6 ± 0.29 | 2.13 ± 0.26 | 9.91 ± 1.05 | 3.22 ± 0.57 | 2.310 |
| n-C32 | 59.42 | 7.85 ± 1.19 | - | - | - | - | - | - | - | 4.59 ± 1.31 | - | 1.817 |
| **n-C34** | 60.156 | 71.78 ± 8.79 | 61.07 ± 10.85 | 85.99 ± 19.85 | 21.98 ± 4.98 | 12 ± 2.47 | 11.31 ± 1.04 | 16.09 ± 1.73 | 6.61 ± 0.94 | - | - | **2.526** |
| **17-C35:1** | 60.301 | - | 33.72 ± 9.9 | 7.09 ± 1.84 | 18.13 ± 4.02 | 16.96 ± 2.9 | 12.8 ± 1.65 | 12.73 ± 0.56 | 5.93 ± 0.97 | - | 4.95 ± 0.86 | **2.651** |
| **n-C35** | 60.792 | 27.15 ± 3.2 | 37.4 ± 17.87 | 108.1 ± 23.4 | - | - | - | 12.67 ± 2.57 | - | 10.75 ± 1.37 | - | **2.834** |
| n-C36 | 61.3 | 255.7 ± 30.86 | 251.75 ± 43.7 | 232.41 ± 70.37 | 82.93 ± 20.28 | 59.84 ± 9.15 | 54.86 ± 6.62 | 55.88 ± 5.41 | 25.57 ± 5.19 | 57.87 ± 4.44 | 17.06 ± 2.57 | 1.803 |
| n-C40 | 62.07 | 15.52 ± 2.06 | - | - | - | 67.69 ± 10.66 | - | - | - | - | - | 2.323 |
| n-C42 | 62.367 | 22.1 ± 2.87 | - | - | - | 16.27 ± 3.69 | - | - | - | - | - | 1.804 |
| n-C43 | 63.052 | 6.77 ± 1.03 | - | 65.51 ± 13.19 | - | - | - | 11.41 ± 1.74 | - | - | - | 2.321 |
| n-C44 | 63.437 | 50.01 ± 6.59 | 62.85 ± 18.48 | 167.4 ± 46.85 | 18.04 ± 4.2 | 207.77 ± 36.87 | 16.46 ± 2.2 | 24.62 ± 2.22 | 6.79 ± 1.13 | 21.3 ± 8.4 | 3.73 ± 0.38 | 2.437 |
| total content | - | 546.61±66.6 | 576.72±104.73 | 1105.41±242.07 | 219.14±51.51 | 415.46±68.32 | 148.86±14.82 | 182.61±11.13 | 63.78±9.84 | 161.11±19.23 | 38.7±4.28 | - |

R.T., retention time of CHCs; M.D.G., mean decrease gini: quantifies variable importance by measuring the improvement in node purity (NodePurity) achieved through splits at each variable in a decision tree.

**Table S4** Permutational multivariate ANOVA (PERMANOVA) was performed to assess the dissimilarities in community structure among fig wasp assemblages associated with *Ficus semicordata*.

| Relationship | **R** | ***p* (adjusted)** |
| --- | --- | --- |
| **Interspecies variation (all species)** | 0.973 | 0.001 |
| **Pairwise Comparisons:** |  |  |
| *C. gravelyi* vs. *P. dunia* | 0.176 | 0.003 |
| *C. gravelyi* vs. *S. cunia* | 0.479 | 0.001 |
| *C. gravelyi* vs. *Apocrypta* sp. | 0.521 | 0.001 |
| *C. gravelyi* vs*. S. trifemmensis* | 0.528 | 0.001 |
| *P. dunia* vs*. S. cunia* | 0.347 | 0.001 |
| *P. dunia* vs. *Apocrypta* sp. | 0.291 | 0.001 |
| *P. dunia* vs*. S. trifemmensis* | 0.466 | 0.001 |
| *S. cunia* vs. *Apocrypta* sp. | 0.504 | 0.001 |
| *S. cunia* vs. *S. trifemmensis* | 0.592 | 0.001 |
| *Apocrypta* sp. vs*. S. trifemmensis* | 0.427 | 0.001 |
| **Intraspecies variation** |  |  |
| *C. gravelyi* | 0.931 | 0.001 |
| *P. dunia* | 0.941 | 0.004 |
| *S. cunia* | 0.899 | 0.001 |
| *Apocrypta* sp. | 0.906 | 0.001 |
| *S. trifemmensis* | 0.977 | 0.001 |

**Table S5** Sex-specific absolute quantities of cuticular hydrocarbon profiles in four dominant fig wasp species associated with *Ficus semicordata* were analyzed across three distinct seasons.

| Compounds | R.T. (min) | *C. gravelyi* | | | | | | *S. cunia* | | | | | | *Apocrypta* sp. | | | | | | *S. trifemmensis* | | | | | |
| --- | --- | --- | --- | --- | --- | --- | --- | --- | --- | --- | --- | --- | --- | --- | --- | --- | --- | --- | --- | --- | --- | --- | --- | --- | --- |
|  |  | Rainy | | Fog-cool | | Dry-hot | | Rainy | | Fog-cool | | Dry-hot | | Rainy | | Fog-cool | | Dry-hot | | Rainy | | Fog-cool | | Dry-hot | |
|  |  | ♀ | *♂* | ♀ | *♂* | ♀ | ♂ | ♀ | *♂* | ♀ | *♂* | ♀ | *♂* | ♀ | *♂* | ♀ | *♂* | ♀ | *♂* | ♀ | *♂* | ♀ | *♂* | ♀ | *♂* |
| n-C7 | 5.361 | - | - | - | - | 0.4 | 0.5 | - | - | - | - | 0.42 | 0.49 | - | - | - | - | 0.38 | 0.38 | - | - | - | - | 0.41 | 0.4 |
| trans-β-Ocimene | 10.004 | - | - | - | - | - | - | - | - | 0.18 | 0.32 | 0.1 | - | - | - | 0.15 | - | - | - | - | - | - | - | - | - |
| n-C13 | 17.136 | - | - | - | - | - | - | - | - | 0.11 | 0.09 | - | - | 0.16 | 0.2 | 0.13 | - | - | - | 0.23 | 0.2 | 0.06 | 0.14 | - | - |
| 2,6,10-triMe-C12 | 35.556 | - | - | - | - | - | - | - | - | - | 0.05 | - | - | 0.07 | 0.04 | - | - | - | - | - | - | - | - | - | - |
| 2-Me-C16-7-ene | 41.57 | - | - | - | - | - | - | - | - | - | - | - | - | - | 0.13 | - | - | - | - | - | - | - | - | - | - |
| n-C17 | 42.119 | 0.18 | - | - | - | - | - | - | - | - | 0.04 | - | - | - | - | - | - | - | - | 0.13 | - | - | - | - | - |
| 2,6-diMe-C17 | 44.11 | 0.09 | - | - | - | - | - | - | - | - | - | - | - | - | - | - | - | - | - | - | - | - | - | - | - |
| 9-C19:1 | 45.342 | - | - | - | - | - | - | - | - | - | - | - | - | - | 0.66 | - | 0.42 | - | - | - | - | - | - | - | - |
| n-C19 | 45.751 | 0.08 | - | - | - | - | - | - | - | - | 0.03 | - | - | 0.09 | - | - | - | - | - | - | - | - | - | - | - |
| 1,19-C20:2 | 44.4 | - | - | - | - | - | - | - | 0.22 | - | - | - | - | - | - | - | - | - | - | - | - | - | - | - | - |
| 2,6-diMe-C18 | 47.365 | - | - | - | - | - | - | - | - | - | 0.17 | - | - | - | - | - | - | - | - | - | - | - | - | - | - |
| C20:1 | 47.699 | - | - | - | - | - | - | - | 0.13 | - | - | - | - | - | - | - | - | - | - | - | - | - | - | - | - |
| 2-Me-C20 | 47.987 | - | - | - | - | - | - | - | - | - | 0.58 | - | - | - | - | 0.12 | 0.64 | - | - | - | - | 0.31 | 0.39 | - | - |
| 10-C21:1 | 48.029 | 1.51 | - | - | - | 0.47 | - | - | - | - | - | - | - | - | - | - | - | - | - | - | - | - | - | - | - |
| n-C21 | 48.448 | - | - | - | - | - | - | - | - | - | 0.08 | - | - | - | - | - | - | - | - | - | - | - | - | - | - |
| 1,9,17-C22:3 | 48.471 | - | 0.37 | - | - | - | - | 0.11 | - | - | - | - | - | - | - | - | - | - | - | - | - | - | - | - | - |
| 3-Me-C21 | 49.391 | - | - | - | - | 2.92 | - | - | - | - | - | - | - | - | - | - | - | - | - | - | - | - | - | - | - |
| 10-C22:1 | 49.415 | - | - | 0.78 | - | - | - | - | - | - | - | - | - | - | - | - | - | - | - | - | - | - | - | - | - |
| 2-Me-C22 | 50.344 | - | - | 1.99 | 5 | - | 4.01 | - | - | - | - | - | 0.28 | 0.53 | 0.59 | - | - | - | - | 0.79 | - | - | - | - | - |
| 4-Me-C22 | 50.352 | 4.24 | 7.84 | - | - | - | - | - | 0.8 | - | - | - | - | - | - | - | - | - | - | - | - | - | - | - | - |
| C23:1 | 50.474 | - | - | - | - | 1.96 | 0.79 | - | - | - | - | - | - | - | - | - | - | - | - | - | - | - | - | - | - |
| 5-Me-C23 | 51.536 | - | - | - | - | - | - | - | - | - | - | - | 0.36 | - | - | - | - | - | - | - | - | - | - | - | - |
| C24:1 | 51.7 | - | - | 1.44 | - | - | - | - | - | - | - | - | - | - | 0.14 | - | - | - | - | - | - | - | - | - | - |
| n-C24 | 52.046 | - | - | - | 0.31 | - | - | - | - | - | 0.42 | - | - | - | - | - | - | - | - | - | - | - | - | - | - |
| C25:1 | 52.734 | - | - | - | - | - | 1.3 | - | - | - | - | - | - | - | 0.4 | - | - | - | - | - | - | - | - | - | - |
| 2-Me-C24 | 52.448 | - | - | - | 0.82 | - | - | - | - | - | - | - | - | - | - | - | - | - | - | 0.52 | - | - | - | - | - |
| n-C25 | 52.822 | - | - | - | 0.43 | - | - | - | 1.32 | - | 0.32 | - | - | - | - | - | - | - | - | 0.3 | - | - | - | - | - |
| 3-Me-C25 | 52.952 | - | - | - | - | - | - | 1.7 | 0.94 | 0.48 | 0.66 | - | - | - | - | - | - | - | - | 0.5 | - | - | - | - | - |
| C26:1 | 53.016 | - | - | - | - | - | - | - | - | - | 0.41 | - | - | - | - | - | - | - | - | - | - | - | - | - | - |
| C27:1 | 55.005 | - | - | - | 0.78 | - | 2.19 | 2.27 | 3.53 | - | - | - | 1.89 | - | - | - | - | - | - | - | - | - | - | 10.31 | - |
| 2-Me-C26 | 54.807 | - | - | - | 0.8 | - | - | - | - | - | - | - | - | 1.09 | - | 0.47 | - | - | - | 1.75 | - | 17.61 | - | - | - |
| n-C27 | 55.728 | - | 2.92 | 1.75 | 1.58 | - | - | - | 1.13 | - | 0.93 | - | - | 0.55 | 0.57 | - | 0.45 | - | - | 2.47 | - | 1.52 | 0.4 | - | - |
| 3-Me-C27 | 56.512 | - | - | - | - | - | - | - | - | - | - | - | - | - | - | - | - | - | - | 5.07 | - | 2.68 | - | 2.4 | - |
| n-C28 | 56.582 | - | - | - | - | - | - | - | - | - | 0.92 | - | - | - | - | - | - | - | - | - | - | - | - | - | - |
| Squalene | 57.645 | 11.14 | 62.34 | 6.67 | 21.58 | 6.16 | 64.62 | 16.56 | 27.02 | 3.19 | 23.66 | 3.53 | 31.8 | 20.62 | 7.62 | 3.67 | 4.93 | 25.17 | 5.93 | - | 1.67 | - | 0.99 | - | 1.48 |
| 2-Me-C28 | 58.124 | 69.67 | 34.56 | 58.37 | 16.63 | 90.68 | 9.28 | 8.43 | 5 | 5.04 | 6.52 | 7.79 | - | 16.22 | 3.19 | 13.78 | 5.33 | 15.23 | - | 28.92 | 2.03 | 21.76 | 4.08 | 27.2 | 2.3 |
| C29:1 | 58.322 | - | 6.07 | - | 3.63 | - | 4.96 | 3.85 | 4.97 | 0.36 | 2.02 | - | 4.5 | 1.55 | 1.36 | 0.32 | - | - | - | - | 1.05 | 6.8 | 0.9 | 88.65 | - |
| n-C29 | 58.596 | 3.18 | 9.75 | 1.6 | 8.28 | - | - | 1.96 | 3.46 | 0.56 | 2.73 | - | - | 2.93 | 1.74 | - | 2.47 | - | - | 4.59 | 2.9 | 3.17 | 3.04 | - | - |
| 15-Me-C29 | 58.935 | - | 6.03 | 2.43 | - | - | - | - | - | - | 0.88 | - | - | 1.72 | - | 1.02 | - | - | - | 11.34 | 1.85 | 6.86 | 1.12 | 11.74 | - |
| n-C31 | 59.328 | - | - | 8.52 | 9.84 | 15.36 | - | - | 4.82 | 2.51 | 3.62 | - | - | 3.6 | 2.13 | 2.51 | 2.35 | - | - | 9.91 | 3.22 | 7.22 | 2.79 | 11.35 | - |
| n-C32 | 59.42 | 7.85 | - | - | - | - | - | - | - | - | - | - | - | - | - | - | - | - | - | 4.59 | - | 2.5 | - | - | - |
| n-C34 | 60.156 | 71.78 | 61.07 | 43.55 | 25.94 | 75.67 | 22.31 | 12 | 11.31 | 4.63 | 7.45 | - | - | 16.09 | 6.61 | 10.71 | 7.67 | - | - | - | - | 27.17 | - | - | 14.61 |
| 17-C35:1 | 60.301 | - | 33.72 | 3.82 | 18.44 | - | 21 | 16.96 | 12.8 | 4.85 | 7.44 | - | 18.29 | 12.73 | 5.93 | 4.25 | 5.31 | 15.17 | - | - | 4.95 | - | 5.42 | - | - |
| n-C35 | 60.792 | 27.15 | 37.4 | 16.77 | 9.62 | 31.08 | - | - | - | 7.03 | 3.69 | - | - | 12.67 | - | 5.54 | - | - | - | 10.75 | - | 16.16 | - | - | - |
| n-C36 | 61.3 | 255.7 | 251.75 | 205.34 | 124.95 | 436.56 | 112.51 | 59.84 | 54.86 | 42.99 | 50.88 | 73.97 | 60.18 | 55.88 | 25.57 | 55.34 | 30.69 | 80.1 | 27.12 | 57.87 | 17.06 | 58.26 | 23.25 | 46.72 | 31.21 |
| n-C40 | 62.07 | 15.52 | - | 11.35 | 7.79 | - | - | 67.69 | - | 37.13 | - | 59.19 | - | - | - | 3.7 | - | - | - | - | - | - | - | - | - |
| n-C42 | 62.367 | 22.1 | - | 15.74 | - | 23.56 | - | 16.27 | - | 8.63 | - | - | - | - | - | - | - | - | - | - | - | - | - | - | - |
| n-C43 | 63.052 | 6.77 | - | - | - | - | - | - | - | - | - | - | - | 11.41 | - | 6.06 | - | - | - | - | - | - | - | - | - |
| n-C44 | 63.437 | 50.01 | 62.85 | 37.36 | 23.3 | 67.31 | 19.82 | 207.77 | 16.46 | 119.83 | 16.98 | 130.03 | - | 24.62 | 6.79 | 12.6 | 7.4 | 26.5 | - | 21.3 | 3.73 | 13 | 4.79 | 12.25 | 6.46 |

R.T., retention time of CHCs.

**Table S6** Permutational multivariate ANOVA (PERMANOVA) was conducted to evaluate the multivariate responses of distinct compound classes to environmental factors. Statistically significant P-values (P < 0.05) are indicated in bold.

| **Species** | **Compounds** | **Environmental variables** | **Female** | | **Male** | |
| --- | --- | --- | --- | --- | --- | --- |
|  |  |  | **R²** | ***P*** | **R²** | ***P*** |
| *Ceratosolen gravelyi* | n-Aikanes | Temperature | 0.151 | **0.002** | 0.266 | **0.001** |
|  |  | Precipitation | 0.337 | **0.001** | 0.175 | **0.002** |
|  | Methylalkanes | Temperature | 0.098 | 0.064 | 0.07 | 0.088 |
|  |  | Precipitation | 0.151 | **0.032** | 0.327 | **0.003** |
|  | Alkenes | Temperature | 0.793 | **0.001** | 0.055 | 0.208 |
|  |  | Precipitation | 0.031 | **0.037** | 0.025 | 0.414 |
|  | Methylalkenes | Temperature | 0.079 | 0.095 | 0.256 | **0.007** |
|  |  | Precipitation | 0.152 | **0.033** | 0.016 | 0.466 |
|  | Total absolute content | Temperature | 0.167 | **0.017** | 0.134 | **0.027** |
|  |  | Precipitation | 0.216 | **0.002** | 0.21 | **0.006** |
| *Sycophaga cunia* | n-Aikanes | Temperature | 0.133 | **0.003** | 0.016 | 0.476 |
|  |  | Precipitation | 0.167 | **0.001** | 0.556 | **0.001** |
|  | Methylalkanes | Temperature | 0.144 | **0.045** | 0.087 | **0.006** |
|  |  | Precipitation | 0.01 | 0.609 | 0.708 | **0.001** |
|  | Alkenes | Temperature | 0.119 | **0.004** | 0.098 | 0.096 |
|  |  | Precipitation | 0.544 | **0.001** | 0.185 | **0.036** |
|  | Methylalkenes | Temperature | 0.289 | **0.001** | 0.039 | 0.369 |
|  |  | Precipitation | 0.296 | **0.001** | 0.114 | 0.117 |
|  | Total absolute content | Temperature | 0.208 | **0.014** | 0.016 | 0.52 |
|  |  | Precipitation | 0.039 | 0.248 | 0.152 | 0.074 |
| *Apocrypta* sp. | n-Aikanes | Temperature | 0.203 | **0.001** | 0.071 | 0.062 |
|  |  | Precipitation | 0.356 | **0.001** | 0.415 | **0.001** |
|  | Methylalkanes | Temperature | 0.028 | 0.389 | 0.245 | **0.002** |
|  |  | Precipitation | 0.001 | 0.844 | 0.403 | **0.001** |
|  | Alkenes | Temperature | 0.643 | **0.001** | 0.027 | 0.194 |
|  |  | Precipitation | 0.122 | **0.001** | 0.667 | **0.001** |
|  | Methylalkenes | Temperature | 0.424 | **0.001** | 0.043 | 0.338 |
|  |  | Precipitation | 0.075 | **0.049** | 0.015 | 0.607 |
|  | Total absolute content | Temperature | 0.302 | **0.001** | 0.053 | 0.175 |
|  |  | Precipitation | 0.005 | 0.68 | 0.339 | **0.003** |
| *Sycoscapter trifemmensis* | n-Aikanes | Temperature | 0.136 | **0.011** | 0.104 | **0.006** |
|  |  | Precipitation | 0.204 | **0.001** | 0.535 | **0.001** |
|  | Methylalkanes | Temperature | 0.081 | **0.041** | 0.243 | **0.001** |
|  |  | Precipitation | 0.006 | 0.584 | 0.277 | **0.001** |
|  | Alkenes | Temperature | 0.011 | 0.058 | 0.156 | **0.005** |
|  |  | Precipitation | 0.93 | **0.001** | 0.562 | **0.001** |
|  | Methylalkenes | Temperature | **-** | **-** | 0.115 | 0.096 |
|  |  | Precipitation | **-** | **-** | 0.00072 | 0.884 |
|  | Total absolute content | Temperature | 0.006 | 0.707 | 0.00002 | 0.979 |
|  |  | Precipitation | 0.1 | 0.137 | 0.221 | **0.021** |
